# Mitochondrial metabolism as a target for acute myeloid leukemia treatment

**DOI:** 10.1186/s40170-021-00253-w

**Published:** 2021-04-21

**Authors:** Svetlana B. Panina, Jingqi Pei, Natalia V. Kirienko

**Affiliations:** Department of BioSciences, Rice University, Houston, TX USA

**Keywords:** Acute myeloid leukemia (AML), Mitochondria, Mitochondrial abnormalities/alterations, Mitochondrial metabolism, Mitocans, Leukemia stem cells, Synergy, Drug combinations

## Abstract

Acute myeloid leukemias (AML) are a group of aggressive hematologic malignancies resulting from acquired genetic mutations in hematopoietic stem cells that affect patients of all ages. Despite decades of research, standard chemotherapy still remains ineffective for some AML subtypes and is often inappropriate for older patients or those with comorbidities. Recently, a number of studies have identified unique mitochondrial alterations that lead to metabolic vulnerabilities in AML cells that may present viable treatment targets. These include mtDNA, dependency on oxidative phosphorylation, mitochondrial metabolism, and pro-survival signaling, as well as reactive oxygen species generation and mitochondrial dynamics. Moreover, some mitochondria-targeting chemotherapeutics and their combinations with other compounds have been FDA-approved for AML treatment. Here, we review recent studies that illuminate the effects of drugs and synergistic drug combinations that target diverse biomolecules and metabolic pathways related to mitochondria and their promise in experimental studies, clinical trials, and existing chemotherapeutic regimens.

## Background

Acute myeloid leukemias (AML) are a group of hematological cancers that involve clonal proliferation of immature myeloid progenitor cells in the bone marrow and peripheral blood. These myeloblasts tend to be intensely proliferative, even to the extent that they can compromise normal blood flow. Proliferation of the myeloblasts generates a bulk of largely non-functional cells, compromising hematopoiesis, leading to neutropenia and increasing vulnerability to infectious disease. AML is one of the most common leukemias to affect adults (~ 120,000 new cases per year worldwide) and is also one of the most lethal. Left untreated, most forms of AML are aggressive and patients can succumb to disease in weeks to a few months [1].

The most common chemotherapy treatment for AML is called induction and consolidation. The first stage, remission induction, is intended to reduce the bulk of the myeloblasts. Induction involves high doses of cytarabine, a nucleoside analog that compromises DNA replication, with an anthracycline antibiotic such as daunorubicin. The induction phase usually lasts for 7 days. After remission has been triggered, treatment moves into the consolidation stage. This step typically involves several 3-day courses of cytarabine, but can also involve hematopoietic stem cell transplantation [2]. Although the precise anti-cancer mechanism of the combination of cytarabine and anthracyclines is still poorly understood, they are believed to function by inflicting DNA damage, which leads to mitochondrial dysfunction and apoptosis [3]. The length of these courses has led to this treatment often being referred to as “7+3” induction and consolidation.

Although induction and consolidation is one of the most effective treatments currently available for AML, it is very hard on patients. This renders the treatment inappropriate for many older patients (65 years or older, who comprise more than half of all newly diagnosed patients), especially those with other contra-indicators, like secondary disease, adverse genotypes, or treatment-resistant cancers [4]. Although there are several options for these patients, including low-dose induction therapy or more targeted treatments, which will be discussed below, most of these treatments are associated with a reduced likelihood of remission (and shorter survival) compared with aggressive chemotherapy. For a large number of patients, only palliative care is available [4]. Problematically, the risk of relapse is high in AML; about one-third of patients who receive even intensive chemotherapy suffer relapse [4].

Despite limited treatment options, virtually no new treatments were approved for AML in the period between 1971 and 2017 [5]. New treatments have been released since, such as a liposomal combination of cytarabine and daunorubicin known as CPX-351, the isocitrate dehydrogenase inhibitor ivosidenib, the tyrosine kinase inhibitor gilteritinib, the sonic hedgehog inhibitor glasdegib, or the Bcl-2 inhibitor venetoclax [6, 7]. Many of these newer treatments target metabolic differences in tumor cells that will be discussed below.

In this review, we discuss studies that explore mitochondrial characteristics of AML myeloblasts and stem cells, in comparison with their normal counterparts, including alterations in metabolism and signaling, mitochondrial respiration, ROS generation and sensitivity, mitochondrial “priming”, and mitophagy. Also, we describe various groups of mitocans—mitochondria-targeting chemotherapeutics—and their effects with regard to biology of AML cells. Finally, this review provides recent evidence on synergistic drug combinations based on mitocans targeting diverse metabolic pathways that have shown promising results by in vitro, in vivo studies, and clinical trials in AML patients.

## Metabolic differences in LSCs

If curing AML was as simple as clearing the rapidly dividing, highly proliferative myeloblasts, treatment would be onerous, but straightforward. Unfortunately, it is not so simple as this. Most patients have a second population of leukemic cells known as leukemic stem cells (LSCs). LSCs share many characteristics with normal hematopoietic stem cells (HSCs), including being CD38^+^ CD34^−^, although LSCs often express other membrane markers that are absent from HSCs (however, the expression of these markers seems to vary among patients) [8]. Like HSCs (and unlike AML myeloblasts), LSCs divide slowly, making conventional anti-proliferative treatments less effective on them. LSCs also provide a reservoir for the re-emergence of the rapidly dividing myeloblasts and are the most common driving force for relapse and treatment resistance, which occurs in about half of all patients who can be treated with aggressive chemotherapy regimens and more than 80% of patients who cannot. Transfer of LSCs to a naïve host can recapitulate the onset of AML [9–12].

Many studies have reported a unique metabolic signature in AML cells [13]. Metabolic reprogramming in leukemic cells transcends the conventional Warburg effect [14], and includes increased glycolysis and elevated ROS levels possibly regulated by PI3K/AKT and mTOR pathways [15, 16]. Correspondingly, a higher level of anabolic pathway precursors, such as intermediates of the citric acid cycle (CAC) and the pentose phosphate pathway (PPP), have been found in AML [14]. High biosynthetic pathway activity is required for the production of the materials essential for cell growth and proliferation. Glutaminolysis is upregulated, and catabolism of this amino acid is a valuable source of both carbon and nitrogen [14]. Glutaminolysis also regulates OxPhos (oxidative phosphorylation) in AML through the production of NADH [14]. However, dysregulation of antioxidants has been found in AML, which potentially promotes leukemogenesis by increasing ROS level [17–19]. Altered lipid metabolism promotes the interaction of AML with bystanding cells, such as adipocytes, activates their lipolysis, and transfers lipids from adipocytes to myeloblasts [20]. Leukemic cells also tend to upregulate fatty acid oxidation via mitochondrial uncoupling [21]. Mutations in cytosolic and mitochondrial isocitrate dehydrogenases (IDH1 and IDH2), resulting in the production of the oncometabolite 2-hydroxyglutarate, are commonly seen in AML cells, and are frequently targeted for therapy, since they limit cellular differentiation and promote leukemogenesis [22, 23].

Part of the difficulty in treating AML is the profound metabolic differences in LSCs [24]. To a first approximation, LSCs retain much of the metabolic profile of healthy HSCs. In addition to dividing more slowly (making them more resistant to nucleoside analogs that disrupt DNA replication), LSCs rely upon oxidative phosphorylation (OxPhos) for ATP generation instead of glycolysis and lactic acid fermentation (the route most tumors use to obtain ATP). This does leave them vulnerable to the production of ROS, which can force cells out of quiescence and trigger programmed cell death pathways. Most ROS are generated in mitochondria via electron transport. LSCs respond to this threat by upregulating autophagy (which is critical for the maintenance of stemness and the elimination of damaged mitochondria that will produce excess ROS) and upregulate the expression of the hypoxic response transcription factor HIF-1α, even in normoxia, to further limit ROS production [25, 26]. Interestingly, LSCs tend to be metabolically inflexible and rely heavily on fatty acid oxidation and glutaminolysis to maintain OxPhos [27, 28].

## Glycolytic disruptions in AML blasts and LSCs

**Fig. 1:**
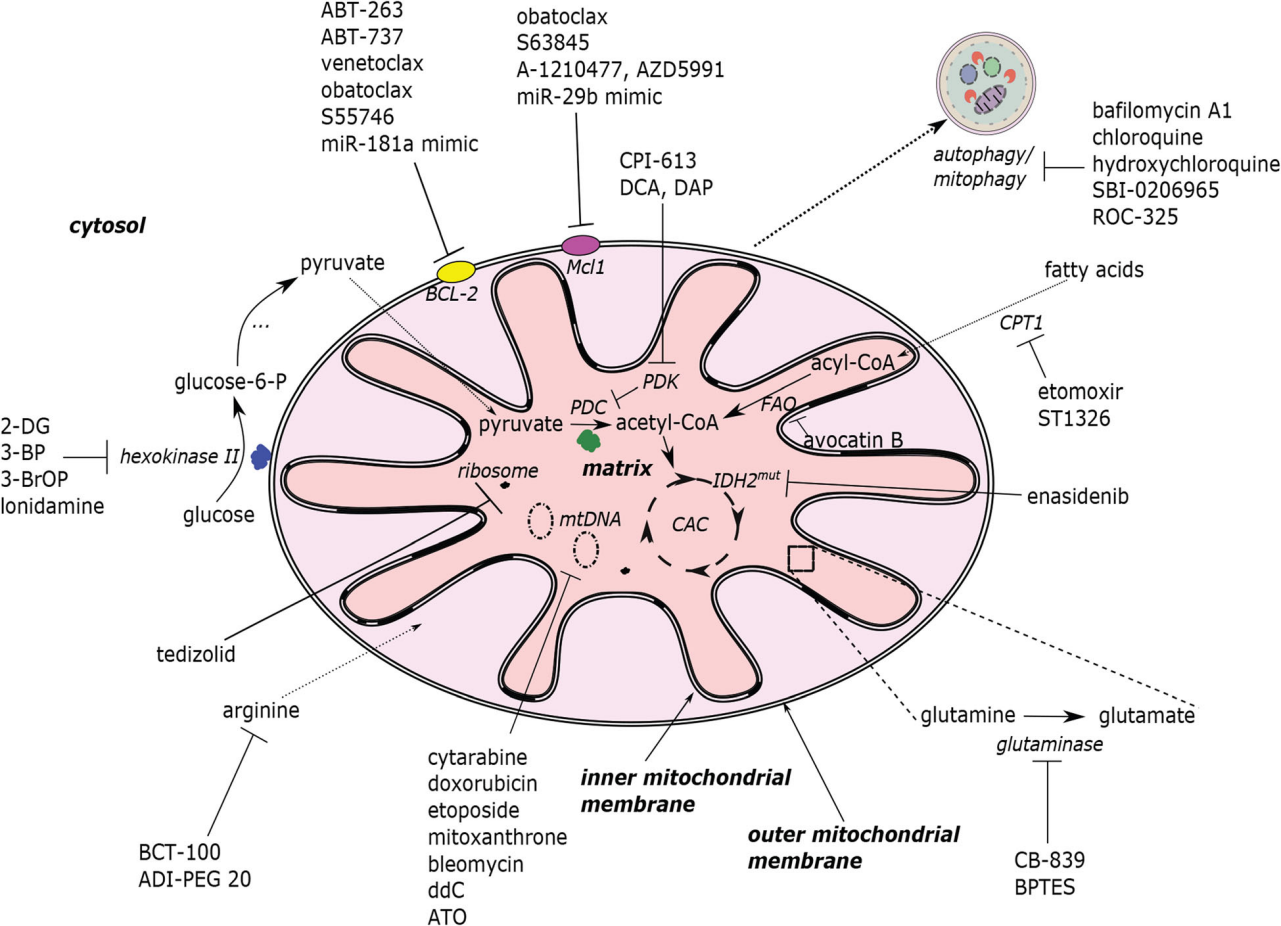
Druggable mitochondrial targets in AML cell and selected pharmacological agents. A depiction of mitochondria, showing important biochemical targets (e.g., the citric acid cycle, mtDNA, mitophagy, etc.) and the drugs that are known to target each. The electron transport chain appears in Fig. 2. α-TOS: (+)alpha-tocopheryl succinate; ANT: adenine nucleotide translocator; ATO: arsenic trioxide; CCCP: carbonyl cyanide m-chlorophenyl hydrazone; CPT1: carnitine O-palmitoyltransferase 1; DAP: 2,2-dichloroacetophenone; DCA: dichloroacetate; ddC: 2′3′-dideoxycytidine; DHODH: dihydroorotate dehydrogenase; FAO: fatty acid oxidation; glucoso-6-P: glucose-6-phosphate; IDH2^mut^: mutant isocitrate dehydrogenase 2; Mcl1: myeloid cell leukemia 1; miR: miRNA; mtDNA: mitochondrial DNA; PDC: pyruvate dehydrogenase complex; PDK: pyruvate dehydrogenase kinase; ROS: reactive oxygen species; SR4,9: dichlorophenyl urea compounds; CAC: citric acid cycle; UCP2: uncoupling protein 2; 2-DG: 2-deoxy-d-glucose; 3-BP: 3-bromopyruvate; 3-BrOP: 3-bromo-2-oxopropionate-1-propyl ester; I–V: Complexes of mitochondrial electron transport chain

**Fig. 2:**
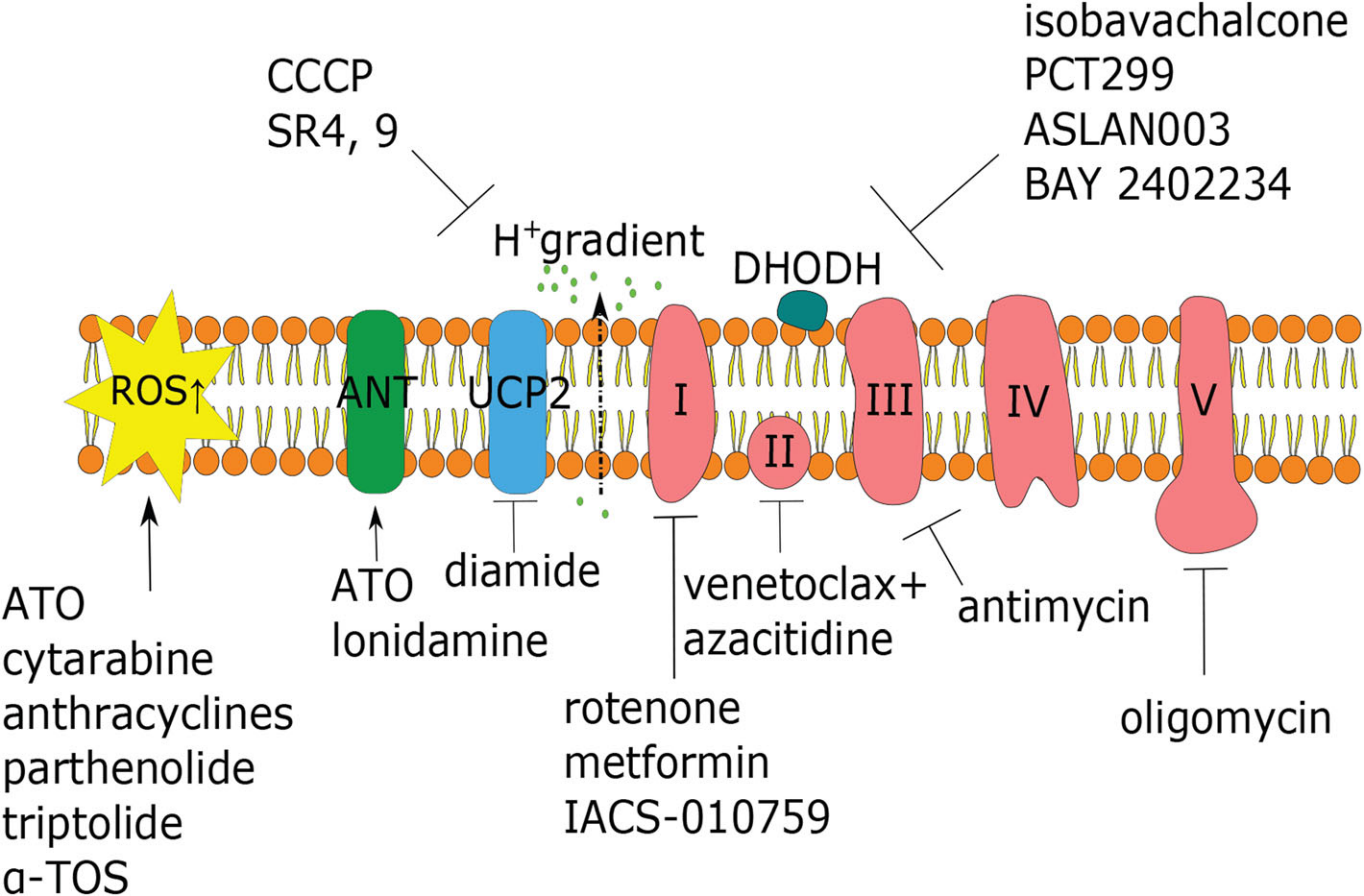
Mitochondrial electron transport chain (ETC) as a therapeutic target in AML. A schematic representation of the ETC, showing the five complexes and potential therapeutic compounds that target each. Also shown is ROS, since the ETC is a major producer of the ROS in the cell by way of electron leak through complexes I and III. ATO: arsenic trioxide; α-TOS: (+)alpha-tocopheryl succinate; ANT: adenine nucleotide translocator; CCCP: carbonyl cyanide m-chlorophenyl hydrazone; DHODH: dihydroorotate dehydrogenase; ROS: reactive oxygen species; SR4,9: dichlorophenyl urea compounds; UCP2: uncoupling protein 2; I–V: complexes of mitochondrial electron transport chain

As noted above, myeloblasts have high glycolytic activity and its anabolic diversions, most importantly the pentose-phosphate pathway, to provide nucleotides, amino acids, and electron carriers, e.g., building blocks that are necessary for rapid proliferation of leukemia cells [29]. The first step of glycolysis, the conversion of glucose to glucose-6-phosphate, is catalyzed by hexokinases. Hexokinase II, the most common version of the enzyme in insulin-sensitive tissues, is a key player in controlling metabolic flux through this pathway. Unsurprisingly, it is also frequently upregulated in cancer cells (reviewed in [30, 31]). One potential method to target hexokinase is to use 3-bromopyruvic acid or 2-deoxy-d-glucose (2-DG), both of which inhibit glucose metabolism [32, 33]. Although targeting hexokinase with 2-DG alone is generally ineffective, it can sensitize AML cells to other drugs that affect mitochondria, including cytarabine, inhibitors of complex I of the ETC (such as rotenone), the mitochondrial uncoupler CCCP, and BH3-mimetic inhibitors of Bcl-2, like ABT-737 [13, 34, 35] (see Figs. 1 and 2 for an overview of druggable mitochondrial targets).

The next rate-limiting, and first committed, step in glycolysis is phosphorylation of 6-phosphofructose by phosphofructokinase-1 (PFK1) to produce fructose 1,6-bisphosphate. PFK1 is allosterically activated by the compound fructose 2,6-bisphosphate, which is overproduced in many cancer types by the overexpression of PFKFB3, a dual function 6-phosphofructo-2-kinase/fructose-2, 6-bisphosphatase that is a therapeutic target itself [36]. Overexpression of PFKFB3, including in leukemia cells, drives increased activity of PFK1, enabling increased glycolytic flux. Computational analysis demonstrated that a novel tumor suppressor, 3-(3-pyridinyl)-1-(4-pyridinyl)-2-propen-1-one (3PO), can competitively inhibit PFKFB3, and decreases intracellular concentrations of fructose 2,6-bisphosphate; this subsequently decreases glycolytic flux in various tumor models [37]. The same group synthesized 73 derivatives of 3PO, one of which (PFK15) was pre-clinically evaluated for targeting resistant hypoxic cancer cells [37]. 3PO was shown to effectively reduce lactate production and cell growth in a leukemia model [38].

A careful analysis of AML patients has revealed a variety of different genetic contributions to disease progression, including some that alter glycolytic activity. One commonly mutated gene is the FMS-like tyrosine kinase 3 gene (known as CD135 or FLT3). Although several amino acid substitutions have been found, the most common category of mutation identified is internal tandem duplication of one or more codons near the transmembrane domain (known as FLT3-ITD). This class of mutations is found in approximately one-third of AML patients and is associated with poor prognosis and increased risk of relapse [39–42]. Oncogenic mutations in FLT3 trigger overactivation of the tyrosine kinase, which promotes several pro-survival effects in cells, including AKT-mediated upregulation of hexokinase—increasing their glycolytic activity [43]. There has been an explosion in treatments available for patients with FLT3 mutations, including a number of tyrosine kinase inhibitors like midostaurin and lestaurtinib (derivatives of staurosporine that target multiple tyrosine kinases), sorafenib, quizartinib, crenolanib, and gilteritinib [44]. Of these, only midostaurin and gilteritinib have received approval from the US Federal Drug Agency, and the latter is the first drug identified to target both internal tandem duplications and tyrosine kinase domain mutations [45]. The metabolic shift caused by the mutation also sensitizes these cells to glycolytic inhibitors like 3-bromopyruvate, which potentiates treatment with tyrosine kinase inhibitors [34, 43].

## Citric acid cycle disruptions in AML blasts and LSCs

Targeting enzymes involved in the flux of pyruvate into the mitochondrial metabolism or citric acid cycle (CAC) is another fruitful anti-leukemia strategy. In the transition between glycolysis and the CAC cycle, pyruvate needs to be decarboxylated and condensed with coenzyme A (CoA) to yield acetyl-CoA that can be combined with oxaloacetate to yield citrate. Acetyl-CoA production requires the pyruvate dehydrogenase complex (PDC), which is comprised of three different enzymes [46]. Interestingly, cancer tissues often exhibit increased expression of PDC kinases, which limit CAC activity, driving pyruvate toward conversion to lactate, with important implications for energy production and modification of the tumor microenvironment. High expression of PDKs in AML patients (particularly PDK3, which is the most active isoform) is a negative prognostic factor for survival [47].

Several synthetic inhibitors of PDKs have been identified, such as Nov3r, AZD7545, Pfz3, radicicol, and CPI-613 [46]. The addition of CPI-613 to conventional chemotherapy is a promising approach for older AML patients and those with poor-risk cytogenetics [48]. Unfortunately, most of these compounds have IC_50_ values in the low- to mid-millimolar range, suggesting that it would be very difficult to deliver appropriate concentrations of these compounds to tumor cells, particularly without unacceptable levels of off-target effects.

Mutations in the isocitrate dehydrogenase (*IDH*) genes of AML patients provided researchers with one of their first hints that mitochondrial metabolism was compromised in AML [30]. This occurs in 20% of AML cases and is associated with grim prognoses (median survival after diagnosis is approximately 6 months) [49]. Commonly, this results in neofunctionalization of IDH1 (cytoplasmic) and IDH2 (mitochondrial) enzymes, allowing them to convert α-ketoglutarate into 2-hydroxyglutarate. This onco-metabolite interferes with cellular metabolism and epigenetic regulation [50] and contributes to mitochondrial DNA instability [51]. It is believed that the ability of 2-hydroxyglutarate to interfere with processes that require α-ketoglutarate, such as histone and DNA demethylation, is also likely to interfere with the prolyl hydroxylases that regulate HIF-1α [52]. As noted above, HIF-1α is well known to play important roles in HSC and LSC maintenance [53]. Recently, ivosidenib and enasidenib, inhibitors of IDH1 and IDH2, have been approved by the US FDA for AML treatment [54]. Mutant IDH proteins can induce a reversible block of differentiation in leukemic cells by preventing IDH enzyme function [55].

## Glutamine: key amino acid for cancer cell viability

Although cancer cells require a wide variety of materials for their survival and growth, at the most basic level this can be reduced to the demand for two nutrients: glucose (for anaerobic glycolysis) and glutamine [56]. Glutamine's uniquely important role in cancer arises from its ability to activate the key mTORC1 pathway (which integrates myriad inputs to regulate metabolic activity, autophagy, and apoptosis) [57, 58]. Glutamine can also be condensed with cysteine and glycine to form glutathione, which supports redox regulation and limits ROS damage; it also provides nitrogen for the synthesis of nucleotides for DNA replication [29, 59].

The key first step in the use of glutamine is carried out by glutaminase, which deaminates glutamine to yield glutamate. Inhibiting glutaminases has proven to be a popular anti-cancer strategy [60]. The most effective current candidate is Calithera’s telaglenastat, also known as CB-839. As might be expected, given the functions of glutamine in cancer, telaglenastat increases susceptibility to redox-targeting therapies like arsenic trioxide and homoharrintonine and improves rates of AML apoptosis [61], while decreasing mTOR signaling [60].

Several AML mutations are known to generate idiosyncratic sensitivities to glutamine metabolism. For example, BPTES, from which CB-839 was derived, exhibits some specificity for *IDH*-mutated AML cells [62]. FLT3-ITD mutations are another example: aberrant FLT3 expression likely increases glutaminolysis. Inhibiting function with the tyrosine kinase inhibitor AC220 (also known as quizartinib) impairs glutamine uptake and glutathione production, hypersensitizing AML cells to oxidative stress [63]. Combination treatment of FLT3-ITD AML cells/primary samples with CB-839 and AC220 consistently resulted in reduced oxygen consumption, increased ROS production, and the activation of apoptosis [64].

## Dependency on mitochondrial mass, mitochondrial respiration, and OxPhos

Both myeloblasts and LSCs have been shown to have increased mitochondrial mass compared with their healthy counterparts, although this difference is more pronounced in myeloblasts than in the CD34^+^CD38^−^ LSCs, further demonstrating the metabolic differences between these populations [65]. Intriguingly, this increase is not associated with a concomitant increase in respiratory function; instead, these cells exhibit a reduced spare reserve capacity, suggesting that their mitochondria are much less efficient [66]. Moreover, we have recently shown that AML cells have reduced coupling efficiency with underlying pre-existing proton leak and enhanced sensitivity to mitochondrial uncouplers compared with normal blood cells [67]. Interestingly, these phenotypes have led to the suggestion that AML progression requires increased mitochondrial biogenesis and OxPhos [67–71].

The importance of OxPhos is further highlighted by the fact that a cytarabine-resistant population of AML cells show enrichment not in LSCs *per se*, but in cells with up-regulated mitochondrial mass, membrane potential, and OxPhos. Importantly, inhibiting the latter improved sensitivity to cytarabine [68]. Quiescent LSCs with a low level of ROS are more reliant on oxidative phosphorylation, as they cannot efficiently utilize glycolysis for energy homeostasis [72]. Consistent with this, the antimicrobial tigecycline, which inhibits mitochondrial translation, selectively kills LSCs (compared with HSCs) by compromising mitochondrial biogenesis in AML cells [65]. Unfortunately, a clinical trial studying the efficacy of intravenous infusion of tigecycline for refractory AML patients failed to show a clinical response, possibly due to the drug's short half-life [73]. Intriguingly, AML cells, including LSCs, are capable of taking up functional mitochondria from other cells in their environment, such as bone marrow cells, increasing their mitochondrial mass, and this phenomenon is thought to contribute to chemoresistance [74, 75]. This mitochondrial transfer increases during chemotherapeutic treatment and was proposed as an additional mechanism that provides AML cells with energy [75]. More specifically, bone marrow mesenchymal stem cells significantly protect leukemic cells from chemotherapy-induced ROS by increasing glutathione availability and utilization, mainly via the glutathione peroxidase system [75].

Mutations in mitochondrial genes that encode complexes I, III, and IV of the electron transport chain (ETC) have been linked to worsened outcomes in AML patients, suggesting that loss of proper function exacerbates disease [76]. However, there is substantial evidence that the complexes of the ETC are viable targets for therapeutic intervention, including in AML. For this reason, various strategies to disable mitochondrial ETC have been investigated in AML.

Among complex I inhibitors, the best known are the anti-diabetic biguanide metformin and the compound IACS-010759 (reviewed in [77, 78]). Metformin stimulates metabolic reprogramming, increasing glycolysis, pentose phosphate pathway, and fatty acid and anaplerotic metabolism and changing mitochondrial gene expression in leukemic cells [79]. Unfortunately, metformin is ineffective as an anti-AML agent on its own. Although it blocks mitochondrial respiration, it barely affects target cell proliferation or viability [77, 80].

In contrast, a more potent complex I inhibitor, IACS-010759, robustly inhibits proliferation and induces apoptosis, likely through a combination of energy depletion and impaired nucleotide biosynthesis due to reduced glutaminolysis [81]. Clinical trials with IACS-010759 for AML patients are still ongoing [81], but there have already been reports of it being used in combination with venetoclax, which showed strong promise at targeting LSCs and myeloblasts using a PDX model [82]. Similarly, we have recently determined that IACS-010759 can synergize with vinorelbine to improve efficacy and specificity, including in primary cells from AML patients [35].

In that same study, we also determined that rotenone, a well-known inhibitor of complex I, could synergize with the glycolytic inhibitor 2-DG. Rotenone has previously been investigated as a potential cancer therapeutic [83], although it was determined that its off-target toxicity and resultant hematopoietic suppression make it inappropriate for use at the dosages required to prevent proliferation [84]. By pairing it with other compounds, the dose required for efficacy can be significantly reduced, increasing the chance that the targeted effects would be more specific [35].

More recently, another drug called mubritinib (also known as TAK-165) was shown to have a strong effect in vivo against AML [85]. Mubritinib, canonically an inhibitor of ERBB2 (a receptor tyrosine kinase of the EGF receptor superfamily), was shown to inhibit the transfer of electrons through the ETC by blocking the function of complex I at ubiquinone [85]. This mechanism is similar to rotenoids and has similar efficacy.

Most attempts to target the ETC have focused on complex I, but some limited research has been performed on other ETC complexes. For example, a combination of venetoclax and azacytidine appears to have a synergistic effect that blocks glutathionylation of succinate dehydrogenase A (a component of complex II) and kills both myeloblasts and LSCs [86]. Targeting complex III may also be productive. For example, antimycin A more effectively limits oxidative phosphorylation and generates increased ROS production in primary AML cells [66]. As will be discussed in the next section, AML cells and LSCs are more sensitive to ROS than their healthy counterparts. Like rotenone, antimycin A is a well-known inhibitor of mitochondrial function; also like rotenone, it is unlikely to be successfully utilized on its own as a therapeutic due to off-target activity. However, it has been effectively combined with a third-generation glycolytic inhibitor, 3-bromo-2-oxopropionoate-1-propyl ester, which serves as a cell-permeable ester of 3-bromopyruvate [87]. This combination potentiated ATP depletion and promoted apoptosis in leukemic cells [87]. It has also shown potential in combination with rapamycin in leukemia and neuroblastoma [87, 88]. Finally, targeting the mitochondrial ATP-synthase (sometimes called complex V) with oligomycin A greatly sensitized leukemia cells to tyrosine kinase inhibitors in FLT3-dependent AML cells, both in vitro and in vivo [89].

Disruption of the ETC on a wider scale is also effective at reducing leukemia cell viability. By targeting the mitochondrial protease ClpP with a beta-lactone inhibitor called A2-32-01, Cole and colleagues demonstrated that this compound was effective at killing leukemia cells with high levels of ClpP expression [90]. Interestingly, this phenotype only appears in approximately half of the leukemic cell lines that were analyzed. Multiple publications have demonstrated that many of the targets of the ClpP protease are members of the ETC complexes [90, 91], perhaps to ensure that the components of the complexes remain in stoichiometric balance.

## Modulation of mitochondrial ROS as AML treatment strategy

**Fig. 3:**
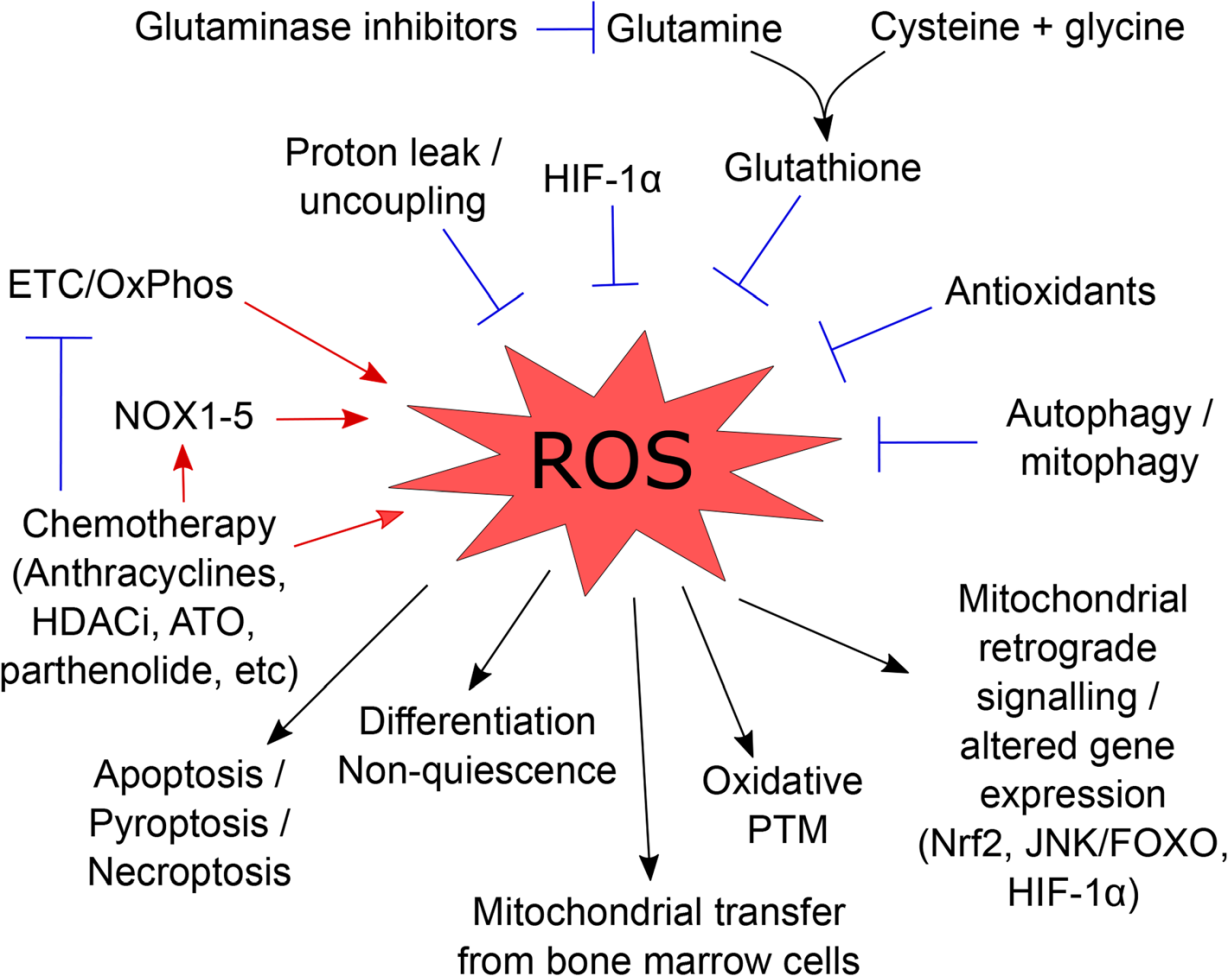
Central role of reactive oxygen species (ROS) in AML biology and treatment. A representation of the wide variety of factors that produce and limit the production of ROS in AML, along with the outcomes of excess ROS production in these cells. ETC: electron transport chain; FOXO: forkhead box protein O; HDACi: histone deacetylases inhibitors; HIF-1α: hypoxia-inducible factor 1α; JNK: c-Jun N-terminal kinase; Nrf2: nuclear factor erythroid 2-related factor 2; NOX: NADH-oxidases; OxPhos: oxidative phosphorylation; PTM: post-translational modifications

The formation of ROS is essential for normal cell physiology (Fig. 3); ROS are generated during mitochondrial oxidative metabolism as well as in response to exposure to xenobiotics, cytokines, and bacterial invaders [92]. But ROS have also long been acknowledged as having a role in cellular signaling [93, 94]. For instance, mitochondrial ROS stimulate signaling pathways promoting tumorigenesis such as JNK/ERK, HIF-1*α*, and mitochondrial biogenesis [94]. ROS have also been shown to regulate protein function (including kinases and phosphatases) via various oxidative post-translational modifications [93].

Superoxide anions (O_2_^−^) are produced as side products from the respiratory chain in mitochondria, by NADH oxidases 1–3 and 5 (NOX), and by other cellular enzymes. The electron transport chain, predominantly complexes I and III, is a major source of superoxide. During oxidative phosphorylation, 1–5% of electrons escape from ETC and produce O_2_^−^ [93]. All NOX family members are transmembrane proteins that use intracellular NADPH to reduce extracellular oxygen to ROS [93]. Interestingly, NOX-derived ROS are linked to activating mutations in FLT3 and Ras: FLT3-ITD mutation in AML causes Akt activation and subsequent stabilization of p22^phox^, a regulatory subunit for NOX1-4 [93, 95]. Moreover, in human AML, NOX2-derived superoxide stimulates bone marrow stromal cells to transfer their mitochondria to AML blasts [96]. Superoxide anions are converted to hydrogen peroxide (H_2_O_2_) by various superoxide dismutases, which are found in several subcellular compartments (the charged nature of superoxide limits its ability to move throughout the cell). Hydrogen peroxide is also produced by NOX4. Other reactive species, such as the short-lived hydroxyl radical (OH^·^), lipid hydroperoxides, peroxynitrite (NO_3_^−^), and hypochlorous acid (HClO), arise by metabolic reactions involving superoxide or H_2_O_2_ [95].

For normal HSCs, ROS present a significant threat, as they can trigger apoptosis, loss of quiescence, or induce differentiation [97]. As noted above, this is also true of LSCs. Metabolic adaptations to limit this sensitivity are likely to emerge and may include increased proton leak down the electrochemical gradient into the mitochondrial matrix [98], mitochondrial uncoupling (abrogation of ATP synthesis in response to Δ_Ψm_) [99], and increased autophagy. These events also promote the Warburg effect and cataplerotic reactions from the CAC and support the shift toward glutaminolysis-dependent fatty acid oxidation (FAO) [100]. This may be why disrupting these events is effective at killing cancer cells. The reduced spare respiratory capacity of AML myeloblasts also makes them unusually vulnerable to oxidative metabolic stress, indicating that increasing ROS may be a viable clinical strategy [66].

The potential for killing AML cells using ROS has been reviewed recently [93]. Redox-based treatments of hematological malignancies can be divided into two different approaches: 1) compounds that stimulate the overproduction of ROS; and 2) compounds that compromise the mitochondrial antioxidant system [101]. It is known that many AML treatments and novel compounds with anti-leukemic activities stimulate ROS production, including cytarabine and anthracyclines (the well-known components of induction and consolidation therapy), histone deacetylase inhibitors, such as vorinostat, and the proteasome inhibitor bortezomib [101, 102]. For example, the cytotoxic effects of the anthracycline doxorubicin are linked to the stimulation of the Fenton reaction that generates lethal hydroxyl radicals from superoxide [103]. The Fenton reaction requires the presence of heavy metals such as iron. One side effect of doxorubicin treatment is the preferential accumulation of iron inside of mitochondria [104]. Contrary to anthracyclines, the HDAC inhibitor vorinostat up-regulates ROS generation in leukemic cells by activating NADH oxidases [105]. Furthermore, the combination of vorinostat and PEITC (β-phenylethyl isothiocyanate), depleting antioxidant glutathione, acts synergistically in AML cells via modulating cellular redox status and H_2_O_2_ accumulation [105].

Arsenic trioxide (ATO) is another interesting example. ATO is a potent ROS inducer and is widely used in combination with all-*trans* retinoic acid (ATRA) to treat acute promyelocytic leukemia (APL), a subtype of AML [106, 107]. ATRA functions in this combination to stimulate differentiation of promyelocytic blast cells, which then spontaneously undergo apoptosis. ATRA also appears to trigger mitochondrial permeability transition and cell death [31]. ATO has replaced anthracycline antibiotics (e.g., daunorubicin, doxorubicin, etc.) as the choice companion drug to treat APL since it exhibits less severe side effects [108]. The cytotoxic effects of ATO on leukemic cells include oxidative stress induction, depolarization of the mitochondrial membrane, DNA damage, and induction of apoptosis [109]. More specifically, ATO increases superoxide generation in leukemia cells by inhibiting mitochondrial respiration upstream of complex IV [110]. Interestingly, in APL cell lines, increased catalase expression has been shown to correlate with ATO resistance [111]. Although ATO has been shown to work in concert with high-dose ascorbate, killing AML and APL blasts while leaving HSCs intact [112], ATO was not effective at treating non-APL forms of AML [113].

There are also several plant-derived compounds that exhibit anti-LSC properties, likely by targeting critical mechanisms of redox balance. These molecules include parthenolide, triptolide, cyclopamine, resveratrol, and avocatin B [114]. Mechanistically, parthenolide and its soluble analogue dimethylamino parthenolide stimulate superoxide anion generation by activating NADH oxidase, followed by activation of the kinase JNK and NK-κB [115]. Interestingly, a rationally designed regimen consisting of parthenolide, 2-deoxy-d-glucose, and temsirolimus has been shown to selectively target LSCs with little to no apparent effect on normal HSCs [116]. The anti-leukemic activity of this regimen is associated with its strong ability to induce oxidative stress without activating the compensatory responses in AML cells [116]. ROS-mediated dimerization of Bax, a pro-apoptotic member of the Bcl-2 protein family, and oxidation of cardiolipin trigger the release of cytochrome *c* into the cytosol, and has been proposed as oxidative stress-based mechanisms of apoptotic activation [117]. Another naturally occurring compound, cyclopamine, inhibits hedgehog signaling and induces apoptosis in AML CD34^+^ blasts [114], and directly inhibits OxPhos in lung tumors [118]. Avocatin B induces ROS-dependent, mitochondria-mediated apoptosis in AML cells, as well as inhibits fatty acid oxidation. It also synergizes with cytarabine/doxorubicin to induce leukemia cell death [114].

Increasing ROS level beyond the capacity of antioxidant defense can cause several types of cell death in AML [101, 102, 119]. Apoptosis is the most common type of cell death resulting from increased ROS production [120]. ROS may activate both mitochondrial (intrinsic) and death receptor (extrinsic) pathways of apoptosis. Mitochondria-derived ROS are able to target mtDNA, disrupt respiratory chain function, lead to loss of mitochondrial membrane potential, impair ATP synthesis, and cause the release of cytochrome *c* due to mitochondrial outer membrane permeabilization [121, 122].

In addition to apoptosis, elevated ROS in AML may also induce ferroptosis [123]. Ferroptosis is an iron-dependent programmed cell death pathway caused by the failure of glutathione-dependent antioxidant defense and unregulated lipid peroxidation [124]. This has implications for AML therapy. For example, low doses of erastin (ferroptosis inducer) enhance the anti-cancer activity of cytarabine or doxorubicin in AML cells [123]. Lastly, necroptosis as a type of cell death is frequently associated with ROS generation in AML [120]. Death receptor activators (TNFα, FasL) can induce mitochondrial and non-mitochondrial ROS generation, followed by activation of ASK1 (apoptosis signal–regulating kinase 1) and p38 MAPK (mitogen-activated protein kinase), which results in caspase-independent AML cell death [121, 125].

One limitation of this approach, at least in theory, is that the increased ROS will contribute to genomic instability that may increase the probability of treatment resistance, with the thought being that any cells that do not develop resistance will perish, leaving only the resistant cells to divide [126]. In addition, AML cells are not the only cells that are very sensitive to ROS; HSCs also exhibit strong sensitivity and it would be difficult to kill the AML cells without also doing considerable, perhaps irreparable, damage to the healthy HSCs.

## Mitochondrial priming and Bcl-2 protein family

Another key requirement for the proliferation of AML LSCs and blasts is the release from apoptotic activation. Generally, in AML, this is achieved by the overexpression of pro-survival Bcl-2 family proteins, including Bcl-X_L_, Mcl-1, and Bcl-2 itself [127]. The anti-apoptotic Bcl-2 proteins are members of a larger superfamily of proteins named after Bcl-2, the founding member. Proteins in the family typically share Bcl-2-like homology domains 1–4 (BH1-BH4), and include both pro- and anti-apoptotic members, although there is a third group, possessing only the BH3 domain, that are also pro-apoptotic [128, 129]. Members of this family trigger apoptosis by intercalating into the outer membrane, increasing its permeability, releasing pro-apoptotic factors, and activating the caspases responsible for a commitment to cell death [130]. Insertion into the membrane appears to, at least partially, depend on whether Bcl-2-family members have oligomerized via their BH3 domains. As reviewed elsewhere [128, 131], conventional cytotoxic chemotherapy often activates apoptosis via mitochondrial permeabilization using Bcl-2-family members.

For these reasons, it has become common to target the BH3 domain of the anti-apoptotic members of the Bcl-2 family with small molecule drugs [132]. The best known and most successful BH3-domain targeting drugs include obatoclax, ABT-737, and an orally available derivative called ABT-263 (also known as navitoclax), which target Bcl-2, Blc-X_L_, and Bcl-W, and ABT-199 (also known as venetoclax), which targets Bcl-2 [86, 132–135]. Venetoclax has become the most clinically effective BH3-targeting drug approved by the FDA for leukemia treatment and has received FDA approval in several different contexts [136–138]. A comprehensive review of venetoclax’s use and function has recently been published [139], so it will not be discussed in depth here.

Although venetoclax has demonstrated encouraging results in targeting Bcl-2, resistance can still develop. The most common cause of this is stabilization of Mcl-1 [140]. This, along with the fact that Mcl-1 is essential for the development and survival of AML cells, has led to the development of selective Mcl-1 inhibitors [141]. Sharon et al. used CRISPR knockout screen to determine that ribosome-targeting antibiotics such as tedizolid can overcome venetoclax resistance by suppressing mitochondrial translation and respiration, and activating the cellular stress response [142]. Moreover, the addition of tedizolid to azacitidine and venetoclax further enhanced the killing of resistant AML cells in vitro and in vivo [142].

Although mitochondrial permeabilization is sometimes considered to be an irreversible commitment, there is evidence that this may be an oversimplification. For example, it has been reported that not all mitochondria undergo permeabilization simultaneously [143, 144]. In addition, it is possible to measure the differences in mitochondrial permeabilization, which revealed that these differences, termed “priming”, are associated with sensitivity to chemotherapy [145]. Interestingly, lower priming is also associated with resistance to chemotherapy [145]. Together, these phenomena suggest that targeting mitochondrial permeability may be an effective method of treating AML in general.

## Mitophagy as a target for AML therapy

Even in healthy cells, the production of ROS is associated with damage to mitochondria. This damage reduces their effective function and can trigger apoptosis or macroautophagic mitochondrial recycling (also known as mitophagy). Under normal circumstances, mitochondria undergo frequent fission and fusion events that, by means yet unknown, sort intact and damaged components so that the former can be retained while the latter are recycled [146].

Although the sorting mechanisms remain unclear, the events coming after have begun to be illuminated. Currently, the best-known regulators of mitophagy are the PTEN-induced kinase 1 (PINK1) and the E3 ubiquitin ligase Parkin, which are conserved at least as far back as the nematode *Caenorhabditis elegans*. While mitochondria are healthy, PINK1 is imported into mitochondria, where it is immediately cleaved by resident proteases [147]. Under stress conditions or loss of mitochondrial membrane potential (i.e., if mitochondrial import is blocked), PINK1 accumulates on the surface of mitochondria, phosphorylating itself and Parkin [148–150]. Once recruited, Parkin catalyzes the ubiquitination that allows mitochondria to be recognized by autophagosomes [146].

Broadly speaking, the idea of autophagy-dependent cell death or, more specifically, mitophagy-dependent cell death, remains controversial [151]. On the one hand, the ability to remove defective mitochondria (mitigating ROS, preventing the activation of apoptosis, and providing building blocks for cell division) makes mitophagy an important tool for leukemic progression. Knockdown of autophagic genes involved in mitochondrial clearance, including BNIP3L/Nix and SQSTM1/p62 also sensitizes cells to mitochondria-targeted therapies [152, 153], arguing that these genes promote cancer cell survival. LSCs have also recently been argued to leverage mitophagy in an attempt to maintain stemness [154]. Mitophagy also promotes survival in the hypoxic conditions that exist in the bone marrow microenvironment [155]. Inhibiting autophagy in these conditions decreased in vivo tumor burden and enhanced apoptosis [155].

In contrast, there is some evidence that mitophagy can limit the growth of cancer cells. For example, inhibiting the activity of complex I in melanoma cells depolarizes the mitochondrial membrane, upregulating ROS, and causes mitophagy-dependent cell death activation [156]. Another report has demonstrated that a trihydroxyphenyl alkone also depolarizes the mitochondrial membrane and triggers autophagic death of melanoma cells [157]. Additionally, loss of autophagy in vivo is associated with a glycolytic shift and more aggressive growth of myelocytes [158]. Sodium selenite, a known activator of mitophagy, triggers programmed cell death in malignant glioma cells via an autophagy-dependent manner [159]. Disruption of autophagy genes is also associated with overproliferation in several solid tumors, further strengthening this connection [160, 161].

Critically, this included HSCs, where deletion of Atg7 or Atg5 resulted in myeloproliferation [158, 162]. Interestingly, these proliferated cells seemed to have lost their stemness and were not able to serve as LSCs, perhaps indicating another key difference between LSCs and myeloblasts. A statistical analysis has demonstrated that mutation of autophagy-related genes occurs more frequently in AML than would be expected by chance [158].

Perhaps most promisingly, there is at least one report that appears to directly target leukemia using activation of mitophagy [163]. The authors of this report demonstrated that FLT3-ITD AML cells were deficient in C_18_ ceramides, which have been associated with apoptosis-independent autophagic cell death [164]. The authors observed that the FLT3-ITD mutation reduced the function of the CerS1 gene (which is responsible for the biosynthesis of C_18_ ceramides) and that disrupting FLT3 improved C_18_ synthesis, which localized to mitochondria, recruited autophagic machinery, and triggered autophagy [163]. Normal markers of apoptosis and necrosis were not observed and pan-caspase and necroptotic inhibitors did not affect FLT3-ITD-targeted rescue. In contrast, bafilomycin A1, which prevents acidification of the autophagolysosome, prevented the cytotoxicity that was triggered by the kinase inhibitors sorafenib, crenolanib, or quizartinib [163]. The report also included evidence that a synthetic ceramide analog could potentiate mitophagy and kill tumor cells by overcoming their resistance to kinase inhibitors. Importantly, this effect was specific to leukemia cells and was observed in a murine PDX AML model, supporting both the potential of this approach and of this particular therapy.

Ultimately, the utility of mitophagy as a tool in the arsenal of anti-cancer treatments may be limited to certain genetic causes of AML or to certain populations of cells, such as myeloblasts or LSCs, but further study of this possibility will be essential to make this determination.

## Conclusions and future perspectives

Over the last three decades, it has become clear that AML cells gain considerable metabolic plasticity during their escape from bone marrow niches and their transitions to proliferating cancer cells. Mitochondria are a central hub for many of these pathways, and the dependency of these cells on mitochondrial function and health is quickly becoming a hallmark of AML, and potentially their Achilles’ heel. It is not surprising that many agents targeting mitochondria and mitochondrial function are currently being investigated in clinical trials or have already been approved by the US FDA for treatment of patients (summarized in Tables 1 and 2, Fig. 1).

**Table 1:** Mitochondria-targeted chemotherapeutics (mitocans) as monotherapy against AML

N	Drugs	Targets/inhibition related to mitochondria	AML subgroup if applicable	Level 1: preclinical (in vitro, PDX)	Level 2: clinical trials/studies in AML patients
**1. DNA-targeted agents/cytotoxic chemotherapy**					
1.1.1	Cytarabine	DNA polymerase, topoisomerase II, incorporation into DNA/RNA	AML	[165]	[166]
1.1.2	Doxorubicin/idarubicin/daunorubicin				[167]
1.1.3	Mitoxanthrone			[168]	[169]
1.1.4	Etoposide			[170]	Phase II [171]
1.2	ddC/alovudine	Mitochondrial DNA polymerase γ, OxPhos	AML	[172, 173]	–
1.3	Bleomycin	mtDNA, OxPhos	AML	[174]	–
**2. Bcl-2 family inhibitors**					
2.1.1	Navitoclax	Bcl-2	AML	[72]	–
2.1.2	Obatoclax			[175]	–
2.1.3	Venetoclax		R/R AML/unfit for intensive therapy	[176, 177]	Phase II [138]
2.2	Obatoclax	Pan Bcl-2	de novo AML	[178]	Phase I/II [179]
			R/R AML		Phase I [180]
2.3.1	S63845/S64315	Mcl1	AML	[181]	Phase I (NCT02979366)
2.3.2	A-1210477			[182]	–
2.3.3	AZD5991		R/R AML	[183]	Phase I/II (NCT03218683)
2.4	α-TOS	Bid cleavage, complex I, ROS production	APL	[184, 185]	–
**3. Agents targeting mitochondrial metabolism**					
3.1.1	2-DG	Hexokinase II	AML, FLT3-ITD AML	[34, 43]	–
3.1.2	3-BP	Hexokinase II, OxPhos, ROS production		[186, 187]	–
3.1.3	3-BrOP	Hexokinase II		[43]	–
3.2	3-PO	6-Phosphofructo-1-kinase	AML	[37]	–
3.3.1	CPI-613	PDK, OxPhos	AML	[188]	Phase I [188]
3.3.2	DAP			[189]	–
3.4	Enasidenib	IDH2^mut^	IDH2^mut^ R/R AML	[190]	Phase I/II [191] FDA-approved
3.5.1	Telaglenastat	Glutaminase	AML	[192]	Phase I (NCT02071927)
3.5.2	BPTES		AML with IDH1/2 mutations	[62]	–
3.6.1	ADI-PEG 20	Arginine depletion	R/R or poor-risk AML	[193]	Phase II (NCT01910012)
3.6.2	BCT-100		Pediatric R/R AML	[194]	Phase I/II (NCT03455140)
3.7	L-asparaginase	Asparagine depletion, glutamine uptake inhibition	AML	[195]	Phase I (NCT02283190)
3.8.1	Etomoxir	FAO (CPT1)	AML	[21]	–
3.8.2	Ranolazine	FAO (3-ketoacyl CoA thiolase)			
3.8.3	ST1326	FAO (CPT1)		[196]	
3.8.4	Avocatin B	FAO, ROS production, cytochrome c release		[197]	
**4. Agents targeting OxPhos and/or mitochondrial biogenesis/respiration**					
4.1	Tigecycline	Mitochondrial translation, mitochondrial biogenesis	AML	[65]	Phase I [73]
4.2.1	Metformin	Complex I, mitochondrial oxygen consumption	AML	[79]	–
4.2.2	IACS-010759		R/R AML	[81]	Phase I (NCT02882321)
4.2.3	Rotenone		AML	[35]	–
4.3	A2-32-01	Mitochondrial protease ClpP, Complex II	AML	[90]	–
4.4	Cysteinase	Complex II	AML	[198]	–
4.5	Antimycin	Complex III	AML	[66]	–
4.6.1	Isobavachalcone	Pyrimidine biosynthesis (DHODH)	AML	[199]	–
4.6.2	PTC299		R/R AML/AML patients unfit for standard therapy	[200]	Phase I (NCT03761069)
4.6.3	ASLAN003			[201]	Phase II (NCT03451084)
4.6.4	BAY 2402234		AML	[202]	Phase I (NCT03404726)
**5. Agents inducing ROS production/targeting MPTP**					
5.1	Arsenic trioxide	ANT, ROS production, MMP, DNA damage	De novo AML, secondary AML, R/R AML	[109]	Phase II [203]
			APL		Phase I/II (NCT00008697)
5.2	Lonidamine	ANT, OxPhos (complex II)	AML	[67, 204, 205]	–
5.3	Parthenolide	ROS production, NF-kB inhibition	AML	[206]	–
5.4	Triptolide (minnelide as a soluble prodrug)	ROS production, Mcl1, MMP	AML	[207]	Phase I/Ib (NCT03760523)
5.5	Resveratrol	NF-kB, apoptosis induction	AML	[208]	–
**6. Mitochondrial uncouplers**					
6.1	CCCP	MMP	AML	[67]	–
6.2	Dichlorophenyl urea (SR4, SR9)	MMP	AML	[209]	–

**Table 2:** Mitochondria-targeted chemotherapeutics (mitocans) in synergistic combinations against AML

N	Drug combination	Targets/inhibition related to mitochondria	AML subgroup if applicable	Level 1: preclinical (in vitro, PDX)	Level 2: clinical trials/studies in AML patients
**1. DNA-targeted combinations/cytotoxic chemotherapy**					
1.1	CPX-351, vyxeos (cytarabine + daunorubicin in liposomal encapsulation at 5:1 synergistic ratio)	mtDNA	AML with myelodysplasia-related changes; therapy-related AML. Can be used to treat elderly patients	[210]	Phase III [211] FDA-approved
1.2	Etoposide + cytarabine + azacitidine	mtDNA	Elderly de novo AML patients	[212]	[213]
1.3	Cytarabine/daunorubicin/idarubicin + HDACi (vorinostat, parabinostat, etc)	mtDNA	R/R AML	[3, 214]	–
			Pediatric AML		Phase I (NCT02676323)
			de novo AML		Phase II [215]
1.4	Etoposide + mitoxanthrone	mtDNA	R/R AML		Phase II [216] [217];
1.5.1	MEC (mitoxanthrone, etoposide, and cytarabine) + sirolimus	mtDNA, mTOR	R/R AML or secondary AML	[218, 219]	Phase I [220]
1.5.2	Cytarabine (consolidation therapy) + everolimus		AML		[221]
1.5.3	Low-dose cytarabine + everolimus		Elderly AML		Phase Ib [222]
1.5.4	Cytarabine + daunorubicin + everolimus		Relapsed AML		Phase I (NCT00544999)
1.6	Cytarabine + ibrutinib	mtDNA, NF-kB	AML	[223]	Phase IIa [224]
1.7	Cytarabine + 2-DG	mtDNA, hexokinase II	AML	[13, 34]	–
**2. Combinations based on apoptosis induction (Bcl-2, Mcl1 inhibition)**					
2.1	Venetoclax + hypometylating agents (e.g., decitabine, azacitidine)	Bcl-2,OxPhos (complex II),amino acid uptake, Nrf2 pathway	De novo/relapsed AML	[86, 225, 226]	Phase Ib [227] FDA-approved
2.2.1	Venetoclax/obatoclax + FAO inhibitors (etomoxir, ranolazine)	Bcl-2, FAO (CPT1a), MPTP	AML	[21]	–
2.2.2	Venetoclax + azacitidine + FAO inhibitors			[28]	
2.3.1	Venetoclax + low-dose cytarabine	Bcl-2, mtDNA	AML patients > 60 y.o. ineligible for induction chemotherapy	[228]	Phase Ib/II [229]; phase III (NCT03069352) FDA-approved
2.3.2	Venetoclax + cytarabine +/- idarubicin		Pediatric R/R AML		Phase I [230]
2.3.3	Venetoclax + cytarabine + daunorubicin; liposome-encapsulated		R/R AML; de novo AML		Phase II (NCT03629171)
2.4	Venetoclax + FLT3-ITD inhibitor (quizartinib)	Bcl-2	AML with FL3-ITD mutation	[231]	Phase Ib/II (NCT03735875)
2.5	Venetoclax + IDH2 mutant inhibitor (enasidenib)	Bcl-2, citric acid cycle	AML with IDH2 mutation R/R AML	[232]	Phase Ib/II (NCT04092179)
2.6.1	Venetoclax + tedizolid	Bcl-2, mitochondrial protein synthesis, OxPhos	AML	[142]	–
2.6.2	Venetoclax + azacitidine + tedizolide				
2.7	Obatoclax + 2-DG	Bcl-2, hexokinase II	AML	[34]	–
2.8	S63845 + S55746	Mcl1, Bcl-2	AML	[233]	–
2.9.1	S63845 + daunorubicin	Mcl1, mtDNA	MLL-AF9 AML	[234]	–
2.9.2	S63845 + venetoclax	Mcl1, Bcl-2			
2.10.1	A-1210477 + venetoclax	Mcl1, Bcl-2	AML	[140]	–
2.10.2	UNBS1450 + venetoclax			[235]	
2.11	AZD5991 + venetoclax	Mcl1, Bcl-2	AML	[183]	Phase I/II (NCT03218683)
2.12	Obatoclax + HDACi	Bcl-2,autophagy induction	AML	[236]	–
**3. Combinations targeting mitochondrial metabolism**					
3.1	CPI-613 + mitoxanthrone + high-dose cytarabine	PDK, mtDNA	R/R AML	[48]	Phase I [48]
3.2	Telaglenastat + venetoclax	Glutaminase, Bcl-2	AML	[59]	–
3.3.1	Telaglenastat + arsenic trioxide	Glutaminase, ROS production, MMP	AML	[61]	–
3.3.2	Telaglenastat + homoharringtonine				
3.4	Telaglenastat + azacitidine	Glutaminase	AML	[237]	Phase I (NCT02071927)
3.5	Telaglenastat + AC220 (FLT3 inhibitor)	Glutaminase, ROS production	FLT3-mutated AML	[238]	–
3.6.1	ADI-PEG 20 (pegylated arginase) + cytarabine	Arginine depletion, mtDNA	AML	[193]	Phase I (NCT02875093)
3.6.2	BCT-100 (pegylated arginase) + cytarabine			[194]	–
3.7.1	Asparaginase + low/high-dose cytarabine	Asparagine depletion, mtDNA	R/R AML/Elderly AML patients > 65 y.o.		Phase II (NCT01810705) [239];
3.7.2	Asparaginase + high-dose cytarabine + mitoxanthrone				[240]
3.8	Etomoxir (FAO inhibitor) + cytarabine	CPT1a, MPTP, mtDNA	AML	[21, 68]	–
3.9.1	Etomoxir + arsenic trioxide	CPT1a, MPTP, ROS production	AML, APL	[241]	–
3.9.2	Etomoxir + arsenic trioxide + 2-DG/lonidamine	CPT1a, MPTP, ROS production, Hexokinase II			
3.10	Avocatin B + cytarabine	FAO, ROS production, mtDNA	AML	[242]	–
**4. Combinations targeting OxPhos**					
4.1.1	Metformin + 2-DG	Complex I, hexokinase II	AML	[79]	–
4.1.2	IACS-010759 + 2-DG			[35]	–
4.1.3	Rotenone + 2-DG				
4.2	Metformin + sorafenib	Complex I, mTOR	FLT3-mutated AML	[243]	–
4.3	Metformin + 6-BT	Complex I, STAT5, glycolysis	FLT3-mutated AML	[244]	–
4.4	Metformin + cytarabine	Complex I, mTOR, mtDNA	R/R AML	[245]	Phase I (NCT01849276)
4.5	Metformin + NSAIDs (diflunisal + diclofenac)	Complex I	AML	[80]	–
4.6	CCCP + 2-DG	MMP, hexokinase II	AML	[35, 67]	–
4.7	IACS-010759 + vinorelbine	Complex I, OxPhos	AML	[35]	–
4.8	IACS-010759 + doxorubicin + cytarabine	Complex I, mtDNA	AML	[246]	–
4.9	Antimycin + 3-BrOP	Complex III, glycolysis, ATP depletion	AML	[87]	–
4.10	Oligomycin + tyrosine kinase inhibitors	Complex V, ROS production	FLT3-mutated AML	[89]	–
4.11	Isobavachalcone + doxorubicin	DHODH, mtDNA	AML	[199]	–
4.12	ASLAN003 + azacitidine	DHODH	AML patients > 60 y.o.		Phase II (NCT03451084)
**5. Combinations inducing ROS generation/targeting mitochondrial membrane complexes**					
5.1	Diamide + doxorubicin	UCP2, mtDNA	AML	[247]	–
5.2	Arsenic trioxide + high-dose ascorbate	ANT, MMP,ROS production	APL (more promising results than in AML)	[112]	Phase II (NCT00184054) [113, 248];
5.3.1	Arsenic trioxide + decitabine/azacitidine	ANT, MMP,ROS production	AML	[249]	Phase II (NCT02190695)
5.3.2	Arsenic trioxide + decitabine/azacitidine + ascorbate				Phase I [250]
5.4.1	Arsenic trioxide + low-dose cytarabine	ANT, MMP, ROS production, mtDNA	AML patients > 60 y.o.		Phase I/II [251]; phase III (NCT00513305) [252];
5.4.2	Arsenic trioxide + high-dose cytarabine + idarubicin		AML patients < 60 y.o.		Phase I [253]
5.5	Arsenic trioxide + mTOR inhibitors (rapamycin)	ANT, MMP, ROS production, mTOR	AML lacking t(15;17) translocation (non-APL)	[254]	–
5.6	Arsenic trioxide + proteasome inhibitor bortezomib	ANT, MMP, ROS production, NF-kB, UPR activation	AML, APL/relapsed APL	[255, 256]	Phase II [257]
5.7.1	Arsenic trioxide + lonidamine	ANT, MMP, ROS production, mTOR, glycolysis	AML	[258]	–
5.7.2	Arsenic trioxide + 3-BP	ANT, MMP, ROS production, glycolysis	AML	[186]	–
5.8	Arsenic trioxide + DCA	ANT, MMP, ROS production, PDK, Mcl1	AML, including FLT3-ITD, R/R AML	[259]	[259]
5.9	Arsenic trioxide + ATRA	ANT, MMP, ROS production	APL	[260]	Phase III [261]
5.10	Parthenolide + 2-DG+ temsirolimus	ROS production, Nrf2, PPP, mTOR, hexokinase II	AML	[116]	–
5.11.1	Parthenolide + ibrutinib	ROS production, NF-kB, mtDNA	AML	[223, 262]	–
5.11.2	Daunorubicin + ibrutinib				
5.12	Triptolide + idarubicin	ROS production, Nrf2, HIF1α	AML	[263]	–
5.13	Resveratrol + HDACi	ROS production, DNA damage	AML	[264]	–
5.14	Cytarabine + PK11195 (PBR ligand)	mtDNA, MPTP	AML	[265]	–
**6. Combinations targeting autophagy/mitophagy**					
6.1.1	Bafilomycin A1 + cytarabine	Autophagy, ROS production, MMP, mtDNA	AML	[266]	–
6.1.2	Chloroquine + cytarabine				
6.1.3	Hydroxychloroquine + cytarabine			[267, 268]	
6.2	Hydroxychloroquine + mitoxanthrone + etoposide	Autophagy, mtDNA	R/R AML		Phase I (NCT02631252)
6.3	Chloroquine + arginase	Autophagy, arginine depletion	AML	[269]	–
6.4	Chloroquine + HDACi (valproic acid/ vorinostat)	Autophagy, accumulation of ubiquitinated proteins	t(8;21)-mutated AML	[270]	–
6.5	ROC-325 + azacitidine	Autophagy	AML	[271]	–
6.6.1	SBI-0206965 + cytarabine	ULK1 (autophagy), ROS production, DNA damage, mtDNA/Bcl-2	AML	[272, 273]	–
6.6.2	SBI-0206965 + venetoclax				
6.6.3	SBI-0206965 + daunorubicin			[274]	
6.7.1	JQ1 + daunorubicin	BET-bromodomain proteins (S100A8/9, BRD4), mtDNA	AML	[275]	–
6.7.2	JQ1 + cytarabine			[276]	
6.8	Birabresib + venetoclax	BET-bromodomain proteins, Bcl-2	AML	[277]	–
6.9	LCL-461 + FLT3-inhibitor crenolanib	Activation of ceramide-dependent mitophagy	AML with FL3-ITD mutation	[163]	–
6.10	TAK-165 + FLT3-inhibitor AC220	Autophagy	AML with FL3-ITD mutation	[278]	–
6.11	Petromurin C + FLT3-inhibitor gilteritinib	Induction of early autophagy and apoptosis, Mcl1	AML with FL3-ITD mutation	[279]	–
**7. Combinations targeting mitochondria-related miRNAs**					
7.1	miR-181a/b mimics + doxorubicin/daunorubicin/cytarabine	Mcl1, Bcl-2, mtDNA	AML	[280–282]	–
7.2	miR-15a/16-1 mimic + arsenic trioxide	UCP2, MMP, cytochrome c release, ROS production	AML	[283]	–
7.3	miR-9 mimic + daunorubicin	EIF5A2, Mcl1, mtDNA	AML	[284]	–
7.4.1	miR-29b mimic + cytarabine	Mcl1, mtDNA	AML	[285]	–
7.4.2	miR-29b mimic + decitabine			[286]	
7.5	Antisense miR-32 + cytarabine	Bim upregulation, mtDNA	AML	[287]	–

One of the most promising future directions in AML therapy is the search for drug combinations with synergistic activity. The utility of this approach has been borne out with the classic example of cytarabine and daunorubicin [210, 211]. These AML-targeted combinations may be comprised of drugs from the same class, such as the pairing of various anthracyclines with cytarabine, or from drugs with different mechanisms of action, such as quizartinib and azacitidine, which inhibit FTL3 and DNA methyltransferase activities, respectively [288, 289]. Since monotherapies are known to result in the development of compensatory mechanisms and/or resistance, the rational design of drug regimens is of great importance. A carefully considered approach can also be more effective and comprehensive than traditional high-throughput searches [116, 290]. For example, the sesquiterpene lactone parthenolide was found to target the redox balance in AML cells, but also led to compensatory activity from the Nrf2 and pentose phosphate pathways [116]. However, by combining parthenolide with the anti-glycolytic 2-deoxy-d-glucose and the mTOR inhibitor temsirolimus, effective AML eradication was achieved [116]. Similarly, classic chemotherapeutics can be paired with novel classes of treatments like autophagic inhibitors or miRNA mimics/antisense to achieve a synergistic therapeutic effect [291, 292].

Where AML was once one of the most lethal and most rapidly developing cancers, the identification and development of effective treatments have made remission a common occurrence. These new approaches, like conventional chemotherapy, can be very effective at inducing remission, even complete remission. Also like conventional treatments, however, they must take into account the metabolic differences between AML myeloblasts and LSCs (such as the differences in energy production, mitochondrial turnover, and sensitivity to ROS) discussed above. As LSCs commonly persist after therapy and are a reservoir for relapse and resistance, it is crucial to continue to investigate these differences and identify new treatments that can specifically target LSCs without causing inappropriate damage to normal HSCs at the same time. Much of our understanding of LSC metabolism has only begun to appear within the last decade, making this an active and developing area of research that promises to lead to even greater improvements in the treatment of AML. When this knowledge is leveraged and these treatment gaps are filled, long-term remission will become commonplace and the promise of effective AML treatments will finally be fulfilled.

## Data Availability

Not applicable.

## Abbreviations

AKT: Protein kinase B (Akt)
α-TOS: (+)Alpha-tocopheryl succinate
ALL: Acute lymphoblastic leukemia
AML: Acute myeloid leukemia
AMPK: AMP-activated protein kinase
ANT: Adenine nucleotide translocator
APL: Acute promyelocytic leukemia
Ara-C: Cytarabine
ATO: Arsenic trioxide
ATRA: All-*trans*-retinoic acid
Bak1: Bcl-2-antagonist/killer 1
Bcl-2: B cell lymphoma 2
BET: Bromodomain and extra-terminal domain
BH(1/2/3/4): Bcl-2 homology domain 1/2/3/4
BRD4: Bromodomain-containing protein 4
CAC: Citric acid cycle
CCCP: Carbonyl cyanide m-chlorophenyl hydrazone
CerS1: Ceramide synthase 1
CPT1: Carnitine O-palmitoyltransferase 1
DAP: 2,2-Dichloroacetophenone
DCA: Dichloroacetate
ddC: 2′3′-Dideoxycytidine
DHODH: Dihydroorotate dehydrogenase
EIF5A2: Eukaryotic translation initiation factor 5A-2
ETC: Electron transport chain
FAO: Fatty acid oxidation
FIS1: Mitochondrial fission 1 protein
FLT3: FMS-like tyrosine kinase 3
GSH: Reduced glutathione
HDACi: Histone deacetylases inhibitors
HIF-1α: Hypoxia-inducible factor 1α
HSC: Hematopoietic stem cells
IDH: Isocytrate dehydrogenase
ITD: Internal tandem duplication
JNK: c-Jun N-terminal kinase
KRAS: K-Ras protein
LSC: Leukemia stem cells
MAPK: Mitogen-activated protein kinase
Mcl1: Myeloid cell leukemia 1
miRNA: MicroRNA
MDR: Multidrug resistance
MMP: Mitochondrial membrane potential
MOMP: Mitochondrial outer membrane permeabilization
MPTP: Mitochondrial permeability transition pore
mtDNA: Mitochondrial DNA
mTORC1: Mammalian target of rapamycin complex 1
NF-kB: Nuclear factor kappa B
NOX: NADH-oxidase
NSAIDs: Nonsteroidal anti-inflammatory drugs
NRAS: Neuroblastoma RAS protein
Nrf2: Nuclear factor erythroid 2-related factor 2
OxPhos: Oxidative phosphorylation
PBMC: Peripheral blood mononuclear cells
PBR: Peripheral benzodiazepine receptor
PDC: Pyruvate dehydrogenase complex
PDK: Pyruvate dehydrogenase kinase
PDX: Patient-derived xenografts
PFK1: Phosphofructokinase-1
PFKFB: Phosphofructokinase-2/fructose bisphosphatase
PINK1: PTEN-induced kinase 1
PPP: Penthose phosphate pathway
ROS: Reactive oxygen species
R/R: Relapsed/refractory AML
TCGA: The Cancer Genome Atlas
UCP: Uncoupling protein
ULK1: Unc-51-like autophagy activating kinase 1
VDAC: Voltage-dependent anion-selective channel
2-DG: 2-Deoxy-d-glucose
2-HG: 2-hydroxyglutarate
3-BP: 3-bromopyruvate
3-BrOP: 3-bromo-2-oxopropionate-1-propyl ester
3-MA: 3-methyladenine
3-PO: 3-(3-pyridinyl)-1-(4-pyridinyl)-2-propen-1-one
4HNE: 4-hydroxy-3-nonenal

## References

[1] Oran B, Weisdorf DJ (2012). Survival for older patients with acute myeloid leukemia: a population-based study. Haematologica..

[2] Dombret H, Gardin C (2016). An update of current treatments for adult acute myeloid leukemia. Blood..

[3] Li Y, Wang Y, Zhou Y, Li J, Chen K, Zhang L, Deng M, Deng S, Li P, Xu B (2017). Cooperative effect of chidamide and chemotherapeutic drugs induce apoptosis by DNA damage accumulation and repair defects in acute myeloid leukemia stem and progenitor cells. Clin Epigenetics..

[4] Döhner H, Estey EH, Amadori S, Appelbaum FR, Büchner T, Burnett AK, Dombret H, Fenaux P, Grimwade D, Larson RA, Lo-Coco F, Naoe T, Niederwieser D, Ossenkoppele GJ, Sanz MA, Sierra J, Tallman MS, Löwenberg B, Bloomfield CD, European LeukemiaNet (2010). Diagnosis and management of acute myeloid leukemia in adults: recommendations from an international expert panel, on behalf of the European LeukemiaNet. Blood..

[5] Pollyea DA, Jordan CT (2019). Why are hypomethylating agents or low-dose cytarabine and venetoclax so effective?. Curr Opin Hematol..

[6] Alfayez M, Kantarjian H, Kadia T, Ravandi-Kashani F, Daver N (2020). CPX-351 (vyxeos) in AML. Leuk Lymphoma..

[7] Kucukyurt S, Eskazan AE (2019). New drugs approved for acute myeloid leukaemia in 2018. Br J Clin Pharmacol..

[8] Hanekamp D, Cloos J, Schuurhuis GJ (2017). Leukemic stem cells: identification and clinical application. Int J Hematol..

[9] Kamel-Reid S, Dick JE (1988). Engraftment of immune-deficient mice with human hematopoietic stem cells. Science..

[10] Kamel-Reid S, Letarte M, Sirard C, Doedens M, Grunberger T, Fulop G, Freedman M, Phillips R, Dick J (1989). A model of human acute lymphoblastic leukemia in immune-deficient SCID mice. Science..

[11] Lapidot T, Pflumio F, Doedens M, Murdoch B, Williams DE, Dick JE (1992). Cytokine stimulation of multilineage hematopoiesis from immature human cells engrafted in SCID mice. Science..

[12] Lapidot T, Sirard C, Vormoor J, Murdoch B, Hoang T, Caceres-Cortes J, Minden M, Paterson B, Caligiuri MA, Dick JE (1994). A cell initiating human acute myeloid leukaemia after transplantation into SCID mice. Nature..

[13] Chen WL, Wang JH, Zhao AH, Xu X, Wang YH, Chen TL, Li JM, Mi JQ, Zhu YM, Liu YF, Wang YY, Jin J, Huang H, Wu DP, Li Y, Yan XJ, Yan JS, Li JY, Wang S, Huang XJ, Wang BS, Chen Z, Chen SJ, Jia W (2014). A distinct glucose metabolism signature of acute myeloid leukemia with prognostic value. Blood..

[14] Castro I, Sampaio-Marques B, Ludovico P. Targeting metabolic reprogramming in acute myeloid leukemia. Cells. 2019;8(9):967. 10.3390/cells8090967 PMC6770240 31450562

[15] Pereira O, Teixeira A, Sampaio-Marques B, Castro I, Girão H, Ludovico P (2018). Signalling mechanisms that regulate metabolic profile and autophagy of acute myeloid leukaemia cells. J Cell Mol Med..

[16] Poulain L, Sujobert P, Zylbersztejn F, Barreau S, Stuani L, Lambert M, Palama TL, Chesnais V, Birsen R, Vergez F, Farge T, Chenevier-Gobeaux C, Fraisse M, Bouillaud F, Debeissat C, Herault O, Récher C, Lacombe C, Fontenay M, Mayeux P, Maciel TT, Portais JC, Sarry JE, Tamburini J, Bouscary D, Chapuis N (2017). High mTORC1 activity drives glycolysis addiction and sensitivity to G6PD inhibition in acute myeloid leukemia cells. Leukemia..

[17] Pei S, Minhajuddin M, Callahan KP, Balys M, Ashton JM, Neering SJ, Lagadinou ED, Corbett C, Ye H, Liesveld JL, O'Dwyer KM, Li Z, Shi L, Greninger P, Settleman J, Benes C, Hagen FK, Munger J, Crooks PA, Becker MW, Jordan CT (2013). Targeting aberrant glutathione metabolism to eradicate human acute myelogenous leukemia cells. J Biol Chem..

[18] Kato M, Minakami H, Kuroiwa M, Kobayashi Y, Oshima S, Kozawa K, Morikawa A, Kimura H (2003). Superoxide radical generation and Mn- and Cu-Zn superoxide dismutases activities in human leukemic cells. Hematol Oncol..

[19] Rasool M, Farooq S, Malik A, Shaukat A, Manan A, Asif M, Sani S, Qazi MH, Kamal MA, Iqbal Z, Hussain A (2015). Assessment of circulating biochemical markers and antioxidative status in acute lymphoblastic leukemia (ALL) and acute myeloid leukemia (AML) patients. Saudi J Biol Sci..

[20] Shafat MS, Oellerich T, Mohr S, Robinson SD, Edwards DR, Marlein CR, Piddock RE, Fenech M, Zaitseva L, Abdul-Aziz A, Turner J, Watkins JA, Lawes M, Bowles KM, Rushworth SA (2017). Leukemic blasts program bone marrow adipocytes to generate a protumoral microenvironment. Blood..

[21] Samudio I, Harmancey R, Fiegl M, Kantarjian H, Konopleva M, Korchin B, Kaluarachchi K, Bornmann W, Duvvuri S, Taegtmeyer H, Andreeff M (2010). Pharmacologic inhibition of fatty acid oxidation sensitizes human leukemia cells to apoptosis induction. J Clin Invest..

[22] Dang L, White DW, Gross S, Bennett BD, Bittinger MA, Driggers EM, Fantin VR, Jang HG, Jin S, Keenan MC, Marks KM, Prins RM, Ward PS, Yen KE, Liau LM, Rabinowitz JD, Cantley LC, Thompson CB, Vander Heiden MG, Su SM (2009). Cancer-associated IDH1 mutations produce 2-hydroxyglutarate. Nature..

[23] Figueroa ME, Abdel-Wahab O, Lu C, Ward PS, Patel J, Shih A, Li Y, Bhagwat N, Vasanthakumar A, Fernandez HF, Tallman MS, Sun Z, Wolniak K, Peeters JK, Liu W, Choe SE, Fantin VR, Paietta E, Löwenberg B, Licht JD, Godley LA, Delwel R, Valk PJM, Thompson CB, Levine RL, Melnick A (2010). Leukemic IDH1 and IDH2 mutations result in a hypermethylation phenotype, disrupt TET2 function, and impair hematopoietic differentiation. Cancer Cell..

[24] Culp-Hill R, D'Alessandro A, Pietras EM. Extinguishing the embers: targeting AML metabolism. Trends Mol Med. 2021;27(4):332–44. 10.1016/j.molmed.2020.10.001 PMC8005405 33121874

[25] Kobayashi CI, Suda T (2012). Regulation of reactive oxygen species in stem cells and cancer stem cells. J Cell Physiol..

[26] Wang Y, Liu Y, Malek SN, Zheng P (2011). Targeting HIF1α eliminates cancer stem cells in hematological malignancies. Cell Stem Cell..

[27] Jones CL, Stevens BM, DʼAlessandro A, Reisz JA, Culp-Hill R, Nemkov T, et al. (2019). Inhibition of amino acid metabolism selectively targets human leukemia stem cells. Cancer Cell..

[28] Jones CL, Stevens BM, Culp-Hill R, Dalessandro A, Krug A, Goosman M (2019). Inhibition of fatty acid metabolism re-sensitizes resistant leukemia stem cells to venetoclax with azacitidine. Blood.

[29] Kreitz J, Schönfeld C, Seibert M, Stolp V, Alshamleh I, Oellerich T, et al. Metabolic Plasticity of Acute Myeloid Leukemia. Cells. 2019;8(8):805. 10.3390/cells8080805 PMC6721808 31370337

[30] Sánchez-Mendoza SE, Rego EM (2017). Targeting the mitochondria in acute myeloid leukemia. Appl Cancer Res..

[31] Fulda S, Galluzzi L, Kroemer G (2010). Targeting mitochondria for cancer therapy. Nat Rev Drug Discov..

[32] Azevedo-Silva J, Queirós O, Baltazar F, Ułaszewski S, Goffeau A, Ko YH, Pedersen PL, Preto A, Casal M (2016). The anticancer agent 3-bromopyruvate: a simple but powerful molecule taken from the lab to the bedside. J Bioenerg Biomembr..

[33] Laussel C, Léon S (2020). Cellular toxicity of the metabolic inhibitor 2-deoxyglucose and associated resistance mechanisms. Biochem Pharmacol..

[34] Larrue C, Saland E, Vergez F, Serhan N, Delabesse E, Mansat-De Mas V (2015). Antileukemic activity of 2-deoxy-d-glucose through inhibition of N-linked glycosylation in acute myeloid leukemia with FLT3-ITD or c-KIT Mutations. Mol Cancer Ther..

[35] Panina SB, Pei J, Baran N, Konopleva M, Kirienko NV (2020). Utilizing synergistic potential of mitochondria-targeting drugs for leukemia therapy. Front Oncol..

[36] Yi M, Ban Y, Tan Y, Xiong W, Li G, Xiang B (2019). 6-Phosphofructo-2-kinase/fructose-2,6-biphosphatase 3 and 4: A pair of valves for fine-tuning of glucose metabolism in human cancer. Mol Metab..

[37] Clem B, Telang S, Clem A, Yalcin A, Meier J, Simmons A, Rasku MA, Arumugam S, Dean WL, Eaton J, Lane A, Trent JO, Chesney J (2008). Small-molecule inhibition of 6-phosphofructo-2-kinase activity suppresses glycolytic flux and tumor growth. Mol Cancer Ther..

[38] Reddy MM, Fernandes MS, Deshpande A, Weisberg E, Inguilizian HV, Abdel-Wahab O, Kung AL, Levine RL, Griffin JD, Sattler M (2012). The JAK2V617F oncogene requires expression of inducible phosphofructokinase/fructose-bisphosphatase 3 for cell growth and increased metabolic activity. Leukemia..

[39] Estey EH (2018). Acute myeloid leukemia: 2019 update on risk-stratification and management. Am J Hematol..

[40] Moarii M, Papaemmanuil E (2017). Classification and risk assessment in AML: integrating cytogenetics and molecular profiling. Hematology..

[41] Papaemmanuil E, Gerstung M, Bullinger L, Gaidzik VI, Paschka P, Roberts ND, Potter NE, Heuser M, Thol F, Bolli N, Gundem G, van Loo P, Martincorena I, Ganly P, Mudie L, McLaren S, O’Meara S, Raine K, Jones DR, Teague JW, Butler AP, Greaves MF, Ganser A, Döhner K, Schlenk RF, Döhner H, Campbell PJ (2016). Genomic classification and prognosis in acute myeloid leukemia. N Engl J Med..

[42] Genomic and epigenomic landscapes of adult de novo acute myeloid leukemia. New England Journal of Medicine. 2013;368(22):2059-74. 10.1056/NEJMoa1301689 PMC3767041 23634996

[43] Ju HQ, Zhan G, Huang A, Sun Y, Wen S, Yang J, Lu WH, Xu RH, Li J, Li Y, Garcia-Manero G, Huang P, Hu Y (2017). ITD mutation in FLT3 tyrosine kinase promotes Warburg effect and renders therapeutic sensitivity to glycolytic inhibition. Leukemia..

[44] Chen Y, Pan Y, Guo Y, Zhao W, Ho WT, Wang J, Xu M, Yang FC, Zhao ZJ (2017). Tyrosine kinase inhibitors targeting FLT3 in the treatment of acute myeloid leukemia. Stem Cell Investig..

[45] Tarver TC, Hill JE, Rahmat L, Perl AE, Bahceci E, Mori K, Smith CC (2020). Gilteritinib is a clinically active FLT3 inhibitor with broad activity against FLT3 kinase domain mutations. Blood Adv..

[46] Stacpoole PW. Therapeutic targeting of the pyruvate dehydrogenase complex/pyruvate dehydrogenase kinase (PDC/PDK) axis in cancer. J Natl Cancer Inst. 2017;109(11):djx071. https://academic.oup.com/jnci/article/109/11/djx071/3871192. 10.1093/jnci/djx071 29059435

[47] Cui L, Cheng Z, Liu Y, Dai Y, Pang Y, Jiao Y, Ke X, Cui W, Zhang Q, Shi J, Fu L (2020). Overexpression of PDK2 and PDK3 reflects poor prognosis in acute myeloid leukemia. Cancer Gene Ther..

[48] Pardee TS, Anderson RG, Pladna KM, Isom S, Ghiraldeli LP, Miller LD, Chou JW, Jin G, Zhang W, Ellis LR, Berenzon D, Howard DS, Hurd DD, Manuel M, Dralle S, Lyerly S, Powell BL (2018). A Phase I Study of CPI-613 in combination with high-dose cytarabine and mitoxantrone for relapsed or refractory acute myeloid leukemia. Clin Cancer Res..

[49] Chifotides HT, Masarova L, Alfayez M, Daver N, Alvarado Y, Jabbour E, Konopleva M, Kantarjian HM, Patel KP, DiNardo CD, Verstovsek S (2020). Outcome of patients with IDH1/2-mutated post-myeloproliferative neoplasm AML in the era of IDH inhibitors. Blood Adv..

[50] Megías-Vericat JE, Ballesta-López O, Barragán E, Montesinos P (2019). IDH1-mutated relapsed or refractory AML: current challenges and future prospects. Blood Lymphat Cancer..

[51] Kingsbury JM, Shamaprasad N, Billmyre RB, Heitman J, Cardenas ME (2016). Cancer-associated isocitrate dehydrogenase mutations induce mitochondrial DNA instability. Hum Mol Genet..

[52] Kickingereder P, Sahm F, Radbruch A, Wick W, Heiland S, Deimling A (2015). IDH mutation status is associated with a distinct hypoxia/angiogenesis transcriptome signature which is non-invasively predictable with rCBV imaging in human glioma. Sci Rep..

[53] Testa U, Labbaye C, Castelli G, Pelosi E (2016). Oxidative stress and hypoxia in normal and leukemic stem cells. Exp Hematol..

[54] Liu X, Gong Y (2019). Isocitrate dehydrogenase inhibitors in acute myeloid leukemia. Biomark Res..

[55] Kats LM, Reschke M, Taulli R, Pozdnyakova O, Burgess K, Bhargava P, Straley K, Karnik R, Meissner A, Small D, Su SM, Yen K, Zhang J, Pandolfi PP (2014). Proto-oncogenic role of mutant IDH2 in leukemia initiation and maintenance. Cell Stem Cell..

[56] Emadi A (2015). Exploiting AML vulnerability: glutamine dependency. Blood..

[57] Jewell JL, Kim YC, Russell RC, Yu FX, Park HW, Plouffe SW, Tagliabracci VS, Guan KL (2015). Metabolism. Differential regulation of mTORC1 by leucine and glutamine. Science..

[58] Cluntun AA, Lukey MJ, Cerione RA, Locasale JW (2017). Glutamine metabolism in cancer: understanding the heterogeneity. Trends Cancer..

[59] Jacque N, Ronchetti AM, Larrue C, Meunier G, Birsen R, Willems L, Saland E, Decroocq J, Maciel TT, Lambert M, Poulain L, Hospital MA, Sujobert P, Joseph L, Chapuis N, Lacombe C, Moura IC, Demo S, Sarry JE, Recher C, Mayeux P, Tamburini J, Bouscary D (2015). Targeting glutaminolysis has antileukemic activity in acute myeloid leukemia and synergizes with BCL-2 inhibition. Blood..

[60] Song M, Kim SH, Im CY, Hwang HJ (2018). Recent Development of small molecule glutaminase inhibitors. Curr Top Med Chem..

[61] Gregory MA, Nemkov T, Park HJ, Zaberezhnyy V, Gehrke S, Adane B, Jordan CT, Hansen KC, D’Alessandro A, DeGregori J (2019). Targeting glutamine metabolism and redox state for leukemia therapy. Clin Cancer Res..

[62] Emadi A, Jun SA, Tsukamoto T, Fathi AT, Minden MD, Dang CV (2014). Inhibition of glutaminase selectively suppresses the growth of primary acute myeloid leukemia cells with IDH mutations. Exp Hematol..

[63] Gregory MA, D'Alessandro A, Alvarez-Calderon F, Kim J, Nemkov T, Adane B (2016). ATM/G6PD-driven redox metabolism promotes FLT3 inhibitor resistance in acute myeloid leukemia. Proc Natl Acad Sci U S A..

[64] Gallipoli P, Giotopoulos G, Tzelepis K, Costa ASH, Vohra S, Medina-Perez P, Basheer F, Marando L, di Lisio L, Dias JML, Yun H, Sasca D, Horton SJ, Vassiliou G, Frezza C, Huntly BJP (2018). Glutaminolysis is a metabolic dependency in FLT3. Blood..

[65] Skrtić M, Sriskanthadevan S, Jhas B, Gebbia M, Wang X, Wang Z (2011). Inhibition of mitochondrial translation as a therapeutic strategy for human acute myeloid leukemia. Cancer Cell..

[66] Sriskanthadevan S, Jeyaraju DV, Chung TE, Prabha S, Xu W, Skrtic M, Jhas B, Hurren R, Gronda M, Wang X, Jitkova Y, Sukhai MA, Lin FH, Maclean N, Laister R, Goard CA, Mullen PJ, Xie S, Penn LZ, Rogers IM, Dick JE, Minden MD, Schimmer AD (2015). AML cells have low spare reserve capacity in their respiratory chain that renders them susceptible to oxidative metabolic stress. Blood..

[67] Panina SB, Baran N, Brasil da Costa FH, Konopleva M, Kirienko NV (2019). A mechanism for increased sensitivity of acute myeloid leukemia to mitotoxic drugs. Cell Death Dis.

[68] Farge T, Saland E, de Toni F, Aroua N, Hosseini M, Perry R, Bosc C, Sugita M, Stuani L, Fraisse M, Scotland S, Larrue C, Boutzen H, Féliu V, Nicolau-Travers ML, Cassant-Sourdy S, Broin N, David M, Serhan N, Sarry A, Tavitian S, Kaoma T, Vallar L, Iacovoni J, Linares LK, Montersino C, Castellano R, Griessinger E, Collette Y, Duchamp O, Barreira Y, Hirsch P, Palama T, Gales L, Delhommeau F, Garmy-Susini BH, Portais JC, Vergez F, Selak M, Danet-Desnoyers G, Carroll M, Récher C, Sarry JE (2017). Chemotherapy-resistant human acute myeloid leukemia cells are not enriched for leukemic stem cells but require oxidative metabolism. Cancer Discov..

[69] Basak NP, Banerjee S (2015). Mitochondrial dependency in progression of acute myeloid leukemia. Mitochondrion..

[70] Schimmer AD, Skrtić M (2012). Therapeutic potential of mitochondrial translation inhibition for treatment of acute myeloid leukemia. Expert Rev Hematol..

[71] Kang MG, Kim YN, Lee JH, Szardenings M, Baek HJ, Kook H, Kim HR, Shin MG (2016). Clinicopathological implications of mitochondrial genome alterations in pediatric acute myeloid leukemia. Ann Lab Med..

[72] Lagadinou ED, Sach A, Callahan K, Rossi RM, Neering SJ, Minhajuddin M, Ashton JM, Pei S, Grose V, O’Dwyer KM, Liesveld JL, Brookes PS, Becker MW, Jordan CT (2013). BCL-2 inhibition targets oxidative phosphorylation and selectively eradicates quiescent human leukemia stem cells. Cell Stem Cell..

[73] Reed GA, Schiller GJ, Kambhampati S, Tallman MS, Douer D, Minden MD, Yee KW, Gupta V, Brandwein J, Jitkova Y, Gronda M, Hurren R, Shamas-Din A, Schuh AC, Schimmer AD (2016). A phase 1 study of intravenous infusions of tigecycline in patients with acute myeloid leukemia. Cancer Med..

[74] Moschoi R, Imbert V, Nebout M, Chiche J, Mary D, Prebet T, Saland E, Castellano R, Pouyet L, Collette Y, Vey N, Chabannon C, Recher C, Sarry JE, Alcor D, Peyron JF, Griessinger E (2016). Protective mitochondrial transfer from bone marrow stromal cells to acute myeloid leukemic cells during chemotherapy. Blood..

[75] Forte D, García-Fernández M, Sánchez-Aguilera A, Stavropoulou V, Fielding C, Martín-Pérez D (2020). Bone Marrow Mesenchymal stem cells support acute myeloid leukemia bioenergetics and enhance antioxidant defense and escape from chemotherapy. Cell Metab.

[76] Wu S, Akhtari M, Alachkar H (2018). Characterization of mutations in the mitochondrial encoded electron transport chain complexes in acute myeloid leukemia. Sci Rep..

[77] Stuani L, Sabatier M, Sarry JE (2019). Exploiting metabolic vulnerabilities for personalized therapy in acute myeloid leukemia. BMC Biol..

[78] Biondani G, Peyron JF (2018). Metformin, an anti-diabetic drug to target leukemia. Front Endocrinol (Lausanne).

[79] Scotland S, Saland E, Skuli N, de Toni F, Boutzen H, Micklow E, Sénégas I, Peyraud R, Peyriga L, Théodoro F, Dumon E, Martineau Y, Danet-Desnoyers G, Bono F, Rocher C, Levade T, Manenti S, Junot C, Portais JC, Alet N, Récher C, Selak MA, Carroll M, Sarry JE (2013). Mitochondrial energetic and AKT status mediate metabolic effects and apoptosis of metformin in human leukemic cells. Leukemia..

[80] Renner K, Seilbeck A, Kauer N, Ugele I, Siska PJ, Brummer C, Bruss C, Decking SM, Fante M, Schmidt A, Hammon K, Singer K, Klobuch S, Thomas S, Gottfried E, Peter K, Kreutz M (2018). Combined metabolic targeting with metformin and the NSAIDs diflunisal and diclofenac induces apoptosis in acute myeloid leukemia cells. Front Pharmacol..

[81] Molina JR, Sun Y, Protopopova M, Gera S, Bandi M, Bristow C, McAfoos T, Morlacchi P, Ackroyd J, Agip ANA, al-Atrash G, Asara J, Bardenhagen J, Carrillo CC, Carroll C, Chang E, Ciurea S, Cross JB, Czako B, Deem A, Daver N, de Groot JF, Dong JW, Feng N, Gao G, Gay J, Do MG, Greer J, Giuliani V, Han J, Han L, Henry VK, Hirst J, Huang S, Jiang Y, Kang Z, Khor T, Konoplev S, Lin YH, Liu G, Lodi A, Lofton T, Ma H, Mahendra M, Matre P, Mullinax R, Peoples M, Petrocchi A, Rodriguez-Canale J, Serreli R, Shi T, Smith M, Tabe Y, Theroff J, Tiziani S, Xu Q, Zhang Q, Muller F, DePinho RA, Toniatti C, Draetta GF, Heffernan TP, Konopleva M, Jones P, di Francesco ME, Marszalek JR (2018). An inhibitor of oxidative phosphorylation exploits cancer vulnerability. Nat Med..

[82] Liu F, Kalpage HA, Wang D, Edwards H, Hüttemann M, Ma J, et al. Cotargeting of mitochondrial complex I and Bcl-2 shows antileukemic activity against acute myeloid leukemia cells reliant on oxidative phosphorylation. Cancers (Basel). 2020;12(9):2400. 10.3390/cancers12092400 PMC7564145 32847115

[83] Matsunaga T, Kudo J, Takahashi K, Dohmen K, Hayashida K, Okamura S, Ishibashi H, Niho Y (1996). Rotenone, a mitochondrial NADH dehydrogenase inhibitor, induces cell surface expression of CD13 and CD38 and apoptosis in HL-60 cells. Leuk Lymphoma..

[84] Heinz S, Freyberger A, Lawrenz B, Schladt L, Schmuck G, Ellinger-Ziegelbauer H (2017). Mechanistic investigations of the mitochondrial complex I inhibitor rotenone in the context of pharmacological and safety evaluation. Sci Rep..

[85] Baccelli I, Gareau Y, Lehnertz B, Gingras S, Spinella JF, Corneau S (2019). Mubritinib targets the electron transport chain complex I and reveals the landscape of OXPHOS dependency in acute myeloid leukemia. Cancer Cell.

[86] Pollyea DA, Stevens BM, Jones CL, Winters A, Pei S, Minhajuddin M, D’Alessandro A, Culp-Hill R, Riemondy KA, Gillen AE, Hesselberth JR, Abbott D, Schatz D, Gutman JA, Purev E, Smith C, Jordan CT (2018). Venetoclax with azacitidine disrupts energy metabolism and targets leukemia stem cells in patients with acute myeloid leukemia. Nat Med..

[87] Akers LJ, Fang W, Levy AG, Franklin AR, Huang P, Zweidler-McKay PA (2011). Targeting glycolysis in leukemia: a novel inhibitor 3-BrOP in combination with rapamycin. Leuk Res..

[88] Levy AG, Zage PE, Akers LJ, Ghisoli ML, Chen Z, Fang W, Kannan S, Graham T, Zeng L, Franklin AR, Huang P, Zweidler-McKay PA (2012). The combination of the novel glycolysis inhibitor 3-BrOP and rapamycin is effective against neuroblastoma. Invest New Drugs..

[89] Alvarez-Calderon F, Gregory MA, Pham-Danis C, DeRyckere D, Stevens BM, Zaberezhnyy V, Hill AA, Gemta L, Kumar A, Kumar V, Wempe MF, Pollyea DA, Jordan CT, Serkova NJ, Graham DK, DeGregori J (2015). Tyrosine kinase inhibition in leukemia induces an altered metabolic state sensitive to mitochondrial perturbations. Clin Cancer Res..

[90] Cole A, Wang Z, Coyaud E, Voisin V, Gronda M, Jitkova Y, Mattson R, Hurren R, Babovic S, Maclean N, Restall I, Wang X, Jeyaraju DV, Sukhai MA, Prabha S, Bashir S, Ramakrishnan A, Leung E, Qia YH, Zhang N, Combes KR, Ketela T, Lin F, Houry WA, Aman A, al-awar R, Zheng W, Wienholds E, Xu CJ, Dick J, Wang JCY, Moffat J, Minden MD, Eaves CJ, Bader GD, Hao Z, Kornblau SM, Raught B, Schimmer AD (2015). Inhibition of the mitochondrial protease ClpP as a therapeutic strategy for human acute myeloid leukemia. Cancer Cell..

[91] Haynes CM, Yang Y, Blais SP, Neubert TA, Ron D (2010). The matrix peptide exporter HAF-1 signals a mitochondrial UPR by activating the transcription factor ZC376.7 in C. elegans. Mol Cell..

[92] Ray PD, Huang BW, Tsuji Y (2012). Reactive oxygen species (ROS) homeostasis and redox regulation in cellular signaling. Cell Signal..

[93] Sillar JR, Germon ZP, DeIuliis GN, Dun MD. The role of reactive oxygen species in acute myeloid leukaemia. Int J Mol Sci. 2019;20(23):6003. 10.3390/ijms20236003 PMC6929020 31795243

[94] Guha M, Avadhani NG (2013). Mitochondrial retrograde signaling at the crossroads of tumor bioenergetics, genetics and epigenetics. Mitochondrion..

[95] Jayavelu AK, Moloney JN, Böhmer FD, Cotter TG (2016). NOX-driven ROS formation in cell transformation of FLT3-ITD-positive AML. Exp Hematol..

[96] Marlein CR, Zaitseva L, Piddock RE, Robinson SD, Edwards DR, Shafat MS, Zhou Z, Lawes M, Bowles KM, Rushworth SA (2017). NADPH oxidase-2 derived superoxide drives mitochondrial transfer from bone marrow stromal cells to leukemic blasts. Blood..

[97] Ludin A, Gur-Cohen S, Golan K, Kaufmann KB, Itkin T, Medaglia C, Lu XJ, Ledergor G, Kollet O, Lapidot T (2014). Reactive oxygen species regulate hematopoietic stem cell self-renewal, migration and development, as well as their bone marrow microenvironment. Antioxid Redox Signal..

[98] Mailloux RJ, McBride SL, Harper ME (2013). Unearthing the secrets of mitochondrial ROS and glutathione in bioenergetics. Trends Biochem Sci..

[99] Brookes PS (2005). Mitochondrial H(+) leak and ROS generation: an odd couple. Free Radic Biol Med..

[100] Vélez J, Hail N, Konopleva M, Zeng Z, Kojima K, Samudio I (2013). Mitochondrial uncoupling and the reprograming of intermediary metabolism in leukemia cells. Front Oncol..

[101] Prieto-Bermejo R, Romo-González M, Pérez-Fernández A, Ijurko C, Hernández-Hernández Á (2018). Reactive oxygen species in haematopoiesis: leukaemic cells take a walk on the wild side. J Exp Clin Cancer Res..

[102] Petruccelli LA, Dupere-Richer D, Pettersson F, Retrouvey H, Skoulikas S, Miller WH (2011). Vorinostat induces reactive oxygen species and DNA damage in acute myeloid leukemia cells. Plos One..

[103] Perillo B, Di Donato M, Pezone A, Di Zazzo E, Giovannelli P, Galasso G (2020). ROS in cancer therapy: the bright side of the moon. Exp Mol Med..

[104] Ichikawa Y, Ghanefar M, Bayeva M, Wu R, Khechaduri A, Naga Prasad SV (2014). Cardiotoxicity of doxorubicin is mediated through mitochondrial iron accumulation. J Clin Invest..

[105] Hu Y, Lu W, Chen G, Zhang H, Jia Y, Wei Y, Yang H, Zhang W, Fiskus W, Bhalla K, Keating M, Huang P, Garcia-Manero G (2010). Overcoming resistance to histone deacetylase inhibitors in human leukemia with the redox modulating compound β-phenylethyl isothiocyanate. Blood..

[106] Zhu HH, Hu J, Lo-Coco F, Jin J (2019). The simpler, the better: oral arsenic for acute promyelocytic leukemia. Blood..

[107] Kumana CR, Mak R, Kwong YL, Gill H (2020). Resurrection of oral arsenic trioxide for treating acute promyelocytic leukaemia: a historical account from bedside to bench to bedside. Front Oncol..

[108] Adès L, Thomas X, Bresler AG, Raffoux E, Spertini O, Vey N, Marchand T, Récher C, Pigneux A, Girault S, Deconinck E, Gardin C, Tournilhac O, Lambert JF, Chevallier P, de Botton S, Lejeune J, Dombret H, Chevret S, Fenaux P (2018). Arsenic trioxide is required in the treatment of newly diagnosed acute promyelocytic leukemia. Analysis of a randomized trial (APL 2006) by the French Belgian Swiss APL group. Haematologica..

[109] Kumar S, Yedjou CG, Tchounwou PB (2014). Arsenic trioxide induces oxidative stress, DNA damage, and mitochondrial pathway of apoptosis in human leukemia (HL-60) cells. J Exp Clin Cancer Res..

[110] Pelicano H, Feng L, Zhou Y, Carew JS, Hileman EO, Plunkett W, Keating MJ, Huang P (2003). Inhibition of mitochondrial respiration: a novel strategy to enhance drug-induced apoptosis in human leukemia cells by a reactive oxygen species-mediated mechanism. J Biol Chem..

[111] Coe E, Schimmer AD (2008). Catalase activity and arsenic sensitivity in acute leukemia. Leuk Lymphoma..

[112] Noguera NI, Pelosi E, Angelini DF, Piredda ML, Guerrera G, Piras E, Battistini L, Massai L, Berardi A, Catalano G, Cicconi L, Castelli G, D’Angiò A, Pasquini L, Graziani G, Fioritoni G, Voso MT, Mastrangelo D, Testa U, Lo-Coco F (2017). High-dose ascorbate and arsenic trioxide selectively kill acute myeloid leukemia and acute promyelocytic leukemia blasts in vitro. Oncotarget..

[113] Aldoss I, Mark L, Vrona J, Ramezani L, Weitz I, Mohrbacher AM, Douer D (2014). Adding ascorbic acid to arsenic trioxide produces limited benefit in patients with acute myeloid leukemia excluding acute promyelocytic leukemia. Ann Hematol..

[114] Siveen KS, Uddin S, Mohammad RM (2017). Targeting acute myeloid leukemia stem cell signaling by natural products. Mol Cancer..

[115] D'Anneo A, Carlisi D, Lauricella M, Puleio R, Martinez R, Di Bella S (2013). Parthenolide generates reactive oxygen species and autophagy in MDA-MB231 cells. A soluble parthenolide analogue inhibits tumour growth and metastasis in a xenograft model of breast cancer. Cell Death Dis..

[116] Pei S, Minhajuddin M, DʼAlessandro A, Nemkov T, Stevens BM, Adane B, et al. (2016). Rational design of a parthenolide-based drug regimen that selectively eradicates acute myelogenous leukemia stem cells. J Biol Chem..

[117] Neuzil J, Wang XF, Dong LF, Low P, Ralph SJ (2006). Molecular mechanism of ʻmitocanʼ-induced apoptosis in cancer cells epitomizes the multiple roles of reactive oxygen species and Bcl-2 family proteins. FEBS Lett..

[118] Kalainayakan SP, Ghosh P, Dey S, Fitzgerald KE, Sohoni S, Konduri PC, Garrossian M, Liu L, Zhang L (2019). Cyclopamine tartrate, a modulator of hedgehog signaling and mitochondrial respiration, effectively arrests lung tumor growth and progression. Sci Rep..

[119] Heasman SA, Zaitseva L, Bowles KM, Rushworth SA, Macewan DJ (2011). Protection of acute myeloid leukaemia cells from apoptosis induced by front-line chemotherapeutics is mediated by haem oxygenase-1. Oncotarget..

[120] Ryter SW, Kim HP, Hoetzel A, Park JW, Nakahira K, Wang X, Choi AMK (2007). Mechanisms of cell death in oxidative stress. Antioxid Redox Signal..

[121] Redza-Dutordoir M, Averill-Bates DA (2016). Activation of apoptosis signalling pathways by reactive oxygen species. Biochim Biophys Acta..

[122] Castelli G, Pelosi E, Testa U. Emerging Therapies for acute myelogenus leukemia patients targeting apoptosis and mitochondrial metabolism. Cancers (Basel). 2019;11(2):260. 10.3390/cancers11020260 PMC6406361 30813354

[123] Yu Y, Xie Y, Cao L, Yang L, Yang M, Lotze MT, Zeh HJ, Kang R, Tang D (2015). The ferroptosis inducer erastin enhances sensitivity of acute myeloid leukemia cells to chemotherapeutic agents. Mol Cell Oncol..

[124] Li J, Cao F, Yin HL, Huang ZJ, Lin ZT, Mao N, Sun B, Wang G (2020). Ferroptosis: past, present and future. Cell Death Dis..

[125] Guilloton F, Jean C, de Thonel A, Laurent G, Quillet-Mary A (2007). Granzyme B induction signalling pathway in acute myeloid leukemia cell lines stimulated by tumor necrosis factor alpha and Fas ligand. Cell Signal..

[126] Sallmyr A, Fan J, Rassool FV (2008). Genomic instability in myeloid malignancies: increased reactive oxygen species (ROS), DNA double strand breaks (DSBs) and error-prone repair. Cancer Lett..

[127] Bradbury DA, Zhu YM, Russell NH (1997). Bcl-2 expression in acute myeloblastic leukaemia: relationship with autonomous growth and CD34 antigen expression. Leuk Lymphoma..

[128] McBride A, Houtmann S, Wilde L, Vigil C, Eischen CM, Kasner M, Palmisiano N (2019). The role of inhibition of apoptosis in acute leukemias and myelodysplastic syndrome. Front Oncol..

[129] Ryan JA, Brunelle JK, Letai A (2010). Heightened mitochondrial priming is the basis for apoptotic hypersensitivity of CD4+ CD8+ thymocytes. Proc Natl Acad Sci U S A..

[130] Kalkavan H, Green DR (2018). MOMP, cell suicide as a BCL-2 family business. Cell Death Differ..

[131] Bohl SR, Bullinger L, Rücker FG. New targeted agents in acute myeloid leukemia: new hope on the rise. Int J Mol Sci. 2019;20(8):1983. 10.3390/ijms20081983 PMC6515298 31018543

[132] Kale J, Osterlund EJ, Andrews DW (2018). BCL-2 family proteins: changing partners in the dance towards death. Cell Death Differ..

[133] Jonas BA, Pollyea DA (2019). How we use venetoclax with hypomethylating agents for the treatment of newly diagnosed patients with acute myeloid leukemia. Leukemia..

[134] Cang S, Iragavarapu C, Savooji J, Song Y, Liu D (2015). ABT-199 (venetoclax) and BCL-2 inhibitors in clinical development. J Hematol Oncol..

[135] Montero J, Letai A (2018). Why do BCL-2 inhibitors work and where should we use them in the clinic?. Cell Death Differ..

[136] DiNardo CD, Pratz K, Pullarkat V, Jonas BA, Arellano M, Becker PS (2019). Venetoclax combined with decitabine or azacitidine in treatment-naive, elderly patients with acute myeloid leukemia. Blood..

[137] Deeks ED (2016). Venetoclax: First Global Approval. Drugs..

[138] Konopleva M, Pollyea DA, Potluri J, Chyla B, Hogdal L, Busman T, McKeegan E, Salem AH, Zhu M, Ricker JL, Blum W, DiNardo CD, Kadia T, Dunbar M, Kirby R, Falotico N, Leverson J, Humerickhouse R, Mabry M, Stone R, Kantarjian H, Letai A (2016). Efficacy and Biological correlates of response in a phase II study of venetoclax monotherapy in patients with acute myelogenous leukemia. Cancer Discov..

[139] Samra B, Konopleva M, Isidori A, Daver N, DiNardo C (2020). Venetoclax-based combinations in acute myeloid leukemia: current evidence and future directions. Front Oncol..

[140] Luedtke DA, Niu X, Pan Y, Zhao J, Liu S, Edwards H, Chen K, Lin H, Taub JW, Ge Y (2017). Inhibition of Mcl-1 enhances cell death induced by the Bcl-2-selective inhibitor ABT-199 in acute myeloid leukemia cells. Signal Transduct Target Ther..

[141] Bolomsky A, Vogler M, Köse MC, Heckman CA, Ehx G, Ludwig H, Caers J (2020). MCL-1 inhibitors, fast-lane development of a new class of anti-cancer agents. J Hematol Oncol..

[142] Sharon D, Cathelin S, Mirali S, Di Trani JM, Yanofsky DJ, Keon KA, et al. Inhibition of mitochondrial translation overcomes venetoclax resistance in AML through activation of the integrated stress response. Sci Transl Med. 2019;11(516):eaax2863. https://pubmed.ncbi.nlm.nih.gov/31666400/. 10.1126/scitranslmed.aax2863 31666400

[143] Rehm M, Huber HJ, Hellwig CT, Anguissola S, Dussmann H, Prehn JH (2009). Dynamics of outer mitochondrial membrane permeabilization during apoptosis. Cell Death Differ..

[144] Bhola PD, Mattheyses AL, Simon SM (2009). Spatial and temporal dynamics of mitochondrial membrane permeability waves during apoptosis. Biophys J..

[145] Ni Chonghaile T, Sarosiek KA, Vo TT, Ryan JA, Tammareddi A, VeG M (2011). Pretreatment mitochondrial priming correlates with clinical response to cytotoxic chemotherapy. Science..

[146] Youle RJ, Narendra DP (2011). Mechanisms of mitophagy. Nat Rev Mol Cell Biol..

[147] Wang N, Zhu P, Huang R, Wang C, Sun L, Lan B, He Y, Zhao H, Gao Y (2020). PINK1: The guard of mitochondria. Life Sci..

[148] Kim Y, Park J, Kim S, Song S, Kwon SK, Lee SH, Kitada T, Kim JM, Chung J (2008). PINK1 controls mitochondrial localization of Parkin through direct phosphorylation. Biochem Biophys Res Commun..

[149] Okatsu K, Oka T, Iguchi M, Imamura K, Kosako H, Tani N, Kimura M, Go E, Koyano F, Funayama M, Shiba-Fukushima K, Sato S, Shimizu H, Fukunaga Y, Taniguchi H, Komatsu M, Hattori N, Mihara K, Tanaka K, Matsuda N (2012). PINK1 autophosphorylation upon membrane potential dissipation is essential for Parkin recruitment to damaged mitochondria. Nat Commun..

[150] Shiba-Fukushima K, Imai Y, Yoshida S, Ishihama Y, Kanao T, Sato S, Hattori N (2012). PINK1-mediated phosphorylation of the Parkin ubiquitin-like domain primes mitochondrial translocation of Parkin and regulates mitophagy. Sci Rep..

[151] Bialik S, Dasari SK, Kimchi A. Autophagy-dependent cell death - where, how and why a cell eats itself to death. J Cell Sci. 2018;131(18):jcs215152. https://pubmed.ncbi.nlm.nih.gov/30237248/. 10.1242/jcs.215152 30237248

[152] Rodrigo R, Mendis N, Ibrahim M, Ma C, Kreinin E, Roma A, Berg S, Blay J, Spagnuolo PA (2019). Knockdown of BNIP3L or SQSTM1 alters cellular response to mitochondria target drugs. Autophagy..

[153] Nguyen TD, Shaid S, Vakhrusheva O, Koschade SE, Klann K, Thölken M, Baker F, Zhang J, Oellerich T, Sürün D, Derlet A, Haberbosch I, Eimer S, Osiewacz HD, Behrends C, Münch C, Dikic I, Brandts CH (2019). Loss of the selective autophagy receptor p62 impairs murine myeloid leukemia progression and mitophagy. Blood..

[154] Pei S, Minhajuddin M, Adane B, Khan N, Stevens BM, Mack SC (2018). AMPK/FIS1-mediated mitophagy is required for self-renewal of human AML stem cells. Cell Stem Cell.

[155] Fay HRS, Dykstra KM, Johnson M, Cronin TL, Lutgen-Dunckley L, Martens BL, Moberg JR, Guzman ML, Wang ES (2019). Mitophagy plays a key role in the anti-leukemic activity of autophagy inhibitors under hypoxia in acute myeloid leukemia. Blood..

[156] Basit F, van Oppen LM, Schöckel L, Bossenbroek HM, van Emst-de Vries SE, Hermeling JC (2017). Mitochondrial complex I inhibition triggers a mitophagy-dependent ROS increase leading to necroptosis and ferroptosis in melanoma cells. Cell Death Dis..

[157] Wang WJ, Wang Y, Chen HZ, Xing YZ, Li FW, Zhang Q, Zhou B, Zhang HK, Zhang J, Bian XL, Li L, Liu Y, Zhao BX, Chen Y, Wu R, Li AZ, Yao LM, Chen P, Zhang Y, Tian XY, Beermann F, Wu M, Han J, Huang PQ, Lin T, Wu Q (2014). Orphan nuclear receptor TR3 acts in autophagic cell death via mitochondrial signaling pathway. Nat Chem Biol..

[158] Watson AS, Riffelmacher T, Stranks A, Williams O, De Boer J, Cain K, et al. Autophagy limits proliferation and glycolytic metabolism in acute myeloid leukemia. Cell Death Discov. 2015;1(1). 10.1038/cddiscovery.2015.8. 10.1038/cddiscovery.2015.8 PMC4641322 26568842

[159] Kim EH, Sohn S, Kwon HJ, Kim SU, Kim MJ, Lee SJ, Choi KS (2007). Sodium selenite induces superoxide-mediated mitochondrial damage and subsequent autophagic cell death in malignant glioma cells. Cancer Res..

[160] Qu X, Yu J, Bhagat G, Furuya N, Hibshoosh H, Troxel A, Rosen J, Eskelinen EL, Mizushima N, Ohsumi Y, Cattoretti G, Levine B (2003). Promotion of tumorigenesis by heterozygous disruption of the beclin 1 autophagy gene. J Clin Invest..

[161] Fimia GM, Stoykova A, Romagnoli A, Giunta L, Di Bartolomeo S, Nardacci R (2007). Ambra1 regulates autophagy and development of the nervous system. Nature..

[162] Mortensen M, Soilleux EJ, Djordjevic G, Tripp R, Lutteropp M, Sadighi-Akha E, Stranks AJ, Glanville J, Knight S, W. Jacobsen SE, Kranc KR, Simon AK (2011). The autophagy protein Atg7 is essential for hematopoietic stem cell maintenance. J Exp Med..

[163] Dany M, Gencer S, Nganga R, Thomas RJ, Oleinik N, Baron KD, Szulc ZM, Ruvolo P, Kornblau S, Andreeff M, Ogretmen B (2016). Targeting FLT3-ITD signaling mediates ceramide-dependent mitophagy and attenuates drug resistance in AML. Blood..

[164] Sentelle RD, Senkal CE, Jiang W, Ponnusamy S, Gencer S, Selvam SP (2012). Ceramide targets autophagosomes to mitochondria and induces lethal mitophagy. Nat Chem Biol..

[165] Butturini A, Santucci MA, Gale RP, Perocco P, Tura S (1990). GM-CSF incubation prior to treatment with cytarabine or doxorubicin enhances drug activity against AML cells in vitro: a model for leukemia chemotherapy. Leuk Res..

[166] Wu D, Duan C, Chen L, Chen S (2017). Efficacy and safety of different doses of cytarabine in consolidation therapy for adult acute myeloid leukemia patients: a network meta-analysis. Sci Rep..

[167] Harousseau JL, Rigal-Huguet F, Hurteloup P, Guy H, Milpied N, Pris J (1989). Treatment of acute myeloid leukemia in elderly patients with oral idarubicin as a single agent. Eur J Haematol..

[168] Bailly JD, Skladanowski A, Bettaieb A, Mansat V, Larsen AK, Laurent G (1997). Natural resistance of acute myeloid leukemia cell lines to mitoxantrone is associated with lack of apoptosis. Leukemia..

[169] Paciucci PA, Cuttner J, Holland JF (1984). Mitoxantrone as a single agent and in combination chemotherapy in patients with refractory acute leukemia. Semin Oncol..

[170] Wang Y, Zhou R, Liliemark J, Gruber A, Lindemalm S, Albertioni F, Liliemark E (2001). In vitro topo II--DNA complex accumulation and cytotoxicity of etoposide in leukaemic cells from patients with acute myelogenous and chronic lymphocytic leukaemia. Leuk Res..

[171] Osby E, Liliemark E, Björkholm M, Liliemark J (2001). Oral etoposide in patients with hematological malignancies: a clinical and pharmacokinetic study. Med Oncol..

[172] Liyanage SU, Hurren R, Voisin V, Bridon G, Wang X, Xu C, MacLean N, Siriwardena TP, Gronda M, Yehudai D, Sriskanthadevan S, Avizonis D, Shamas-Din A, Minden MD, Bader GD, Laposa R, Schimmer AD (2017). Leveraging increased cytoplasmic nucleoside kinase activity to target mtDNA and oxidative phosphorylation in AML. Blood..

[173] Yehudai D, Liyanage SU, Hurren R, Rizoska B, Albertella M, Gronda M, Jeyaraju DV, Wang X, Barghout SH, MacLean N, Siriwardena TP, Jitkova Y, Targett-Adams P, Schimmer AD (2019). The thymidine dideoxynucleoside analog, alovudine, inhibits the mitochondrial DNA polymerase γ, impairs oxidative phosphorylation and promotes monocytic differentiation in acute myeloid leukemia. Haematologica..

[174] Yeung M, Hurren R, Nemr C, Wang X, Hershenfeld S, Gronda M, Liyanage S, Wu Y, Augustine J, Lee EA, Spagnuolo PA, Southall N, Chen C, Zheng W, Jeyaraju DV, Minden MD, Laposa R, Schimmer AD (2015). Mitochondrial DNA damage by bleomycin induces AML cell death. Apoptosis..

[175] Konopleva M, Contractor R, Tsao T, Samudio I, Ruvolo PP, Kitada S, Deng X, Zhai D, Shi YX, Sneed T, Verhaegen M, Soengas M, Ruvolo VR, McQueen T, Schober WD, Watt JC, Jiffar T, Ling X, Marini FC, Harris D, Dietrich M, Estrov Z, McCubrey J, May WS, Reed JC, Andreeff M (2006). Mechanisms of apoptosis sensitivity and resistance to the BH3 mimetic ABT-737 in acute myeloid leukemia. Cancer Cell..

[176] Souers AJ, Leverson JD, Boghaert ER, Ackler SL, Catron ND, Chen J, Dayton BD, Ding H, Enschede SH, Fairbrother WJ, Huang DCS, Hymowitz SG, Jin S, Khaw SL, Kovar PJ, Lam LT, Lee J, Maecker HL, Marsh KC, Mason KD, Mitten MJ, Nimmer PM, Oleksijew A, Park CH, Park CM, Phillips DC, Roberts AW, Sampath D, Seymour JF, Smith ML, Sullivan GM, Tahir SK, Tse C, Wendt MD, Xiao Y, Xue JC, Zhang H, Humerickhouse RA, Rosenberg SH, Elmore SW (2013). ABT-199, a potent and selective BCL-2 inhibitor, achieves antitumor activity while sparing platelets. Nat Med..

[177] Pan R, Hogdal LJ, Benito JM, Bucci D, Han L, Borthakur G, Cortes J, DeAngelo DJ, Debose LK, Mu H, Döhner H, Gaidzik VI, Galinsky I, Golfman LS, Haferlach T, Harutyunyan KG, Hu J, Leverson JD, Marcucci G, Müschen M, Newman R, Park E, Ruvolo PP, Ruvolo V, Ryan J, Schindela S, Zweidler-McKay P, Stone RM, Kantarjian H, Andreeff M, Konopleva M, Letai AG (2014). Selective BCL-2 inhibition by ABT-199 causes on-target cell death in acute myeloid leukemia. Cancer Discov..

[178] Konopleva M, Watt J, Contractor R, Tsao T, Harris D, Estrov Z, Bornmann W, Kantarjian H, Viallet J, Samudio I, Andreeff M (2008). Mechanisms of antileukemic activity of the novel Bcl-2 homology domain-3 mimetic GX15-070 (obatoclax). Cancer Res..

[179] Schimmer AD, Raza A, Carter TH, Claxton D, Erba H, DeAngelo DJ (2014). A multicenter phase I/II study of obatoclax mesylate administered as a 3- or 24-hour infusion in older patients with previously untreated acute myeloid leukemia. Plos One..

[180] Schimmer AD, O'Brien S, Kantarjian H, Brandwein J, Cheson BD, Minden MD, Yee K, Ravandi F, Giles F, Schuh A, Gupta V, Andreeff M, Koller C, Chang H, Kamel-Reid S, Berger M, Viallet J, Borthakur G (2008). A phase I study of the pan bcl-2 family inhibitor obatoclax mesylate in patients with advanced hematologic malignancies. Clin Cancer Res..

[181] Kotschy A, Szlavik Z, Murray J, Davidson J, Maragno AL, Le Toumelin-Braizat G (2016). The MCL1 inhibitor S63845 is tolerable and effective in diverse cancer models. Nature..

[182] Wang Q, Hao S (2019). A-1210477, a selective MCL-1 inhibitor, overcomes ABT-737 resistance in AML. Oncol Lett..

[183] Tron AE, Belmonte MA, Adam A, Aquila BM, Boise LH, Chiarparin E, Cidado J, Embrey KJ, Gangl E, Gibbons FD, Gregory GP, Hargreaves D, Hendricks JA, Johannes JW, Johnstone RW, Kazmirski SL, Kettle JG, Lamb ML, Matulis SM, Nooka AK, Packer MJ, Peng B, Rawlins PB, Robbins DW, Schuller AG, Su N, Yang W, Ye Q, Zheng X, Secrist JP, Clark EA, Wilson DM, Fawell SE, Hird AW (2018). Discovery of Mcl-1-specific inhibitor AZD5991 and preclinical activity in multiple myeloma and acute myeloid leukemia. Nat Commun..

[184] dos Santos GA, Abreu e Lima RS, Pestana CR, Lima AS, Scheucher PS, Thomé CH (2012). (+)α-Tocopheryl succinate inhibits the mitochondrial respiratory chain complex I and is as effective as arsenic trioxide or ATRA against acute promyelocytic leukemia in vivo. Leukemia..

[185] Yamamoto S, Tamai H, Ishisaka R, Kanno T, Arita K, Kobuchi H, Utsumi K (2000). Mechanism of alpha-tocopheryl succinate-induced apoptosis of promyelocytic leukemia cells. Free Radic Res..

[186] Calviño E, Estañ MC, Sánchez-Martín C, Brea R, de Blas E (2014). Boyano-Adánez MeC, et al. Regulation of death induction and chemosensitizing action of 3-bromopyruvate in myeloid leukemia cells: energy depletion, oxidative stress, and protein kinase activity modulation. J Pharmacol Exp Ther..

[187] Chen Z, Zhang H, Lu W, Huang P (2009). Role of mitochondria-associated hexokinase II in cancer cell death induced by 3-bromopyruvate. Biochim Biophys Acta..

[188] Pardee TS, Lee K, Luddy J, Maturo C, Rodriguez R, Isom S, Miller LD, Stadelman KM, Levitan D, Hurd D, Ellis LR, Harrelson R, Manuel M, Dralle S, Lyerly S, Powell BL (2014). A phase I study of the first-in-class antimitochondrial metabolism agent, CPI-613, in patients with advanced hematologic malignancies. Clin Cancer Res..

[189] Qin L, Tian Y, Yu Z, Shi D, Wang J, Zhang C, Peng R, Chen X, Liu C, Chen Y, Huang W, Deng W (2016). Targeting PDK1 with dichloroacetophenone to inhibit acute myeloid leukemia (AML) cell growth. Oncotarget..

[190] Yen K, Travins J, Wang F, David MD, Artin E, Straley K, Padyana A, Gross S, DeLaBarre B, Tobin E, Chen Y, Nagaraja R, Choe S, Jin L, Konteatis Z, Cianchetta G, Saunders JO, Salituro FG, Quivoron C, Opolon P, Bawa O, Saada V, Paci A, Broutin S, Bernard OA, de Botton S, Marteyn BS, Pilichowska M, Xu YX, Fang C, Jiang F, Wei W, Jin S, Silverman L, Liu W, Yang H, Dang L, Dorsch M, Penard-Lacronique V, Biller SA, Su SSM (2017). AG-221, a first-in-class therapy targeting acute myeloid leukemia harboring oncogenic IDH2 mutations. Cancer Discov..

[191] Stein EM, DiNardo CD, Pollyea DA, Fathi AT, Roboz GJ, Altman JK (2017). Enasidenib in mutant. Blood..

[192] Matre P, Velez J, Jacamo R, Qi Y, Su X, Cai T, Chan SM, Lodi A, Sweeney SR, Ma H, Davis RE, Baran N, Haferlach T, Su X, Flores ER, Gonzalez D, Konoplev S, Samudio I, DiNardo C, Majeti R, Schimmer AD, Li W, Wang T, Tiziani S, Konopleva M (2016). Inhibiting glutaminase in acute myeloid leukemia: metabolic dependency of selected AML subtypes. Oncotarget..

[193] Miraki-Moud F, Ghazaly E, Ariza-McNaughton L, Hodby KA, Clear A, Anjos-Afonso F, Liapis K, Grantham M, Sohrabi F, Cavenagh J, Bomalaski JS, Gribben JG, Szlosarek PW, Bonnet D, Taussig DC (2015). Arginine deprivation using pegylated arginine deiminase has activity against primary acute myeloid leukemia cells in vivo. Blood..

[194] Mussai F, Egan S, Higginbotham-Jones J, Perry T, Beggs A, Odintsova E, Loke J, Pratt G, U KP, Lo A, Ng M, Kearns P, Cheng P, de Santo C (2015). Arginine dependence of acute myeloid leukemia blast proliferation: a novel therapeutic target. Blood..

[195] Willems L, Jacque N, Jacquel A, Neveux N, Maciel TT, Lambert M (2013). Inhibiting glutamine uptake represents an attractive new strategy for treating acute myeloid leukemia. Blood..

[196] Ricciardi MR, Mirabilii S, Allegretti M, Licchetta R, Calarco A, Torrisi MR, Foà R, Nicolai R, Peluso G, Tafuri A (2015). Targeting the leukemia cell metabolism by the CPT1a inhibition: functional preclinical effects in leukemias. Blood..

[197] Lee EA, Angka L, Rota SG, Hanlon T, Mitchell A, Hurren R, Wang XM, Gronda M, Boyaci E, Bojko B, Minden M, Sriskanthadevan S, Datti A, Wrana JL, Edginton A, Pawliszyn J, Joseph JW, Quadrilatero J, Schimmer AD, Spagnuolo PA (2015). Targeting mitochondria with avocatin B induces selective leukemia cell death. Cancer Res..

[198] Jones CL, Stevens BM, DʼAlessandro A, Culp-Hill R, Reisz JA, Pei S, et al. (2019). Cysteine depletion targets leukemia stem cells through inhibition of electron transport complex II. Blood..

[199] Wu D, Wang W, Chen W, Lian F, Lang L, Huang Y, Xu Y, Zhang N, Chen Y, Liu M, Nussinov R, Cheng F, Lu W, Huang J (2018). Pharmacological inhibition of dihydroorotate dehydrogenase induces apoptosis and differentiation in acute myeloid leukemia cells. Haematologica..

[200] Cao L, Weetall M, Trotta C, Cintron K, Ma J, Kim MJ, Furia B, Romfo C, Graci JD, Li W, du J, Sheedy J, Hedrick J, Risher N, Yeh S, Qi H, Arasu T, Hwang S, Lennox W, Kong R, Petruska J, Moon YC, Babiak J, Davis TW, Jacobson A, Almstead NG, Branstrom A, Colacino JM, Peltz SW (2019). Targeting of hematologic malignancies with PTC299, A novel potent inhibitor of dihydroorotate dehydrogenase with favorable pharmaceutical properties. Mol Cancer Ther..

[201] Zhou J, Quah JY, Ng Y, Chooi JY, Toh SH, Lin B, et al. ASLAN003, a potent dihydroorotate dehydrogenase inhibitor for differentiation of acute myeloid leukemia. Haematologica. 2020;105(9):2286–97. 10.3324/haematol.2019.230482 PMC7556493 33054053

[202] Christian S, Merz C, Evans L, Gradl S, Seidel H, Friberg A, Eheim A, Lejeune P, Brzezinka K, Zimmermann K, Ferrara S, Meyer H, Lesche R, Stoeckigt D, Bauser M, Haegebarth A, Sykes DB, Scadden DT, Losman JA, Janzer A (2019). The novel dihydroorotate dehydrogenase (DHODH) inhibitor BAY 2402234 triggers differentiation and is effective in the treatment of myeloid malignancies. Leukemia..

[203] Parmar S, Rundhaugen LM, Boehlke L, Riley M, Nabhan C, Raji A, Frater JL, Tallman MS (2004). Phase II trial of arsenic trioxide in relapsed and refractory acute myeloid leukemia, secondary leukemia and/or newly diagnosed patients at least 65 years old. Leuk Res..

[204] Belzacq AS, El Hamel C, Vieira HL, Cohen I, Haouzi D, Métivier D (2001). Adenine nucleotide translocator mediates the mitochondrial membrane permeabilization induced by lonidamine, arsenite and CD437. Oncogene..

[205] Guo L, Shestov AA, Worth AJ, Nath K, Nelson DS, Leeper DB, Glickson JD, Blair IA (2016). Inhibition of mitochondrial complex II by the anticancer agent lonidamine. J Biol Chem..

[206] Guzman ML, Rossi RM, Karnischky L, Li X, Peterson DR, Howard DS, Jordan CT (2005). The sesquiterpene lactone parthenolide induces apoptosis of human acute myelogenous leukemia stem and progenitor cells. Blood..

[207] Giri B, Gupta VK, Yaffe B, Modi S, Roy P, Sethi V, Lavania SP, Vickers SM, Dudeja V, Banerjee S, Watts J, Saluja A (2019). Pre-clinical evaluation of Minnelide as a therapy for acute myeloid leukemia. J Transl Med..

[208] Estrov Z, Shishodia S, Faderl S, Harris D, Van Q, Kantarjian HM (2003). Resveratrol blocks interleukin-1beta-induced activation of the nuclear transcription factor NF-kappaB, inhibits proliferation, causes S-phase arrest, and induces apoptosis of acute myeloid leukemia cells. Blood..

[209] Figarola JL, Weng Y, Lincoln C, Horne D, Rahbar S (2012). Novel dichlorophenyl urea compounds inhibit proliferation of human leukemia HL-60 cells by inducing cell cycle arrest, differentiation and apoptosis. Invest New Drugs..

[210] Tardi P, Johnstone S, Harasym N, Xie S, Harasym T, Zisman N, Harvie P, Bermudes D, Mayer L (2009). In vivo maintenance of synergistic cytarabine:daunorubicin ratios greatly enhances therapeutic efficacy. Leuk Res..

[211] Lancet JE, Uy GL, Cortes JE, Newell LF, Lin TL, Ritchie EK, Stuart RK, Strickland SA, Hogge D, Solomon SR, Stone RM, Bixby DL, Kolitz JE, Schiller GJ, Wieduwilt MJ, Ryan DH, Hoering A, Banerjee K, Chiarella M, Louie AC, Medeiros BC (2018). CPX-351 (cytarabine and daunorubicin) liposome for injection versus conventional cytarabine plus daunorubicin in older patients with newly diagnosed secondary acute myeloid leukemia. J Clin Oncol..

[212] Füller M, Klein M, Schmidt E, Rohde C, Göllner S, Schulze I (2015). 5-azacytidine enhances efficacy of multiple chemotherapy drugs in AML and lung cancer with modulation of CpG methylation. Int J Oncol..

[213] Onec B, Okutan H, Albayrak M, Can ES, Aslan V, Koluman BU, Kosemehmetoglu OS, Albayrak A (2018). Combination therapy with azacitidine, etoposide, and cytarabine in the treatment of elderly acute myeloid leukemia patients: A single center experience. J Cancer Res Ther..

[214] Maiso P, Colado E, Ocio EM, Garayoa M, Martín J, Atadja P, Pandiella A, San-Miguel JF (2009). The synergy of panobinostat plus doxorubicin in acute myeloid leukemia suggests a role for HDAC inhibitors in the control of DNA repair. Leukemia..

[215] Garcia-Manero G, Tambaro FP, Bekele NB, Yang H, Ravandi F, Jabbour E, Borthakur G, Kadia TM, Konopleva MY, Faderl S, Cortes JE, Brandt M, Hu Y, McCue D, Newsome WM, Pierce SR, de Lima M, Kantarjian HM (2012). Phase II trial of vorinostat with idarubicin and cytarabine for patients with newly diagnosed acute myelogenous leukemia or myelodysplastic syndrome. J Clin Oncol..

[216] Ho AD, Lipp T, Ehninger G, Illiger HJ, Meyer P, Freund M, Hunstein W (1988). Combination of mitoxantrone and etoposide in refractory acute myelogenous leukemia--an active and well-tolerated regimen. J Clin Oncol..

[217] Im A, Amjad A, Agha M, Raptis A, Hou JZ, Farah R, Lim S, Sehgal A, Dorritie KA, Redner RL, McLaughlin B, Shuai Y, Duggal S, Boyiadzis M (2016). Mitoxantrone and etoposide for the treatment of acute myeloid leukemia patients in first relapse. Oncol Res..

[218] Janus A, Linke A, Cebula B, Robak T, Smolewski P (2009). Rapamycin, the mTOR kinase inhibitor, sensitizes acute myeloid leukemia cells, HL-60 cells, to the cytotoxic effect of arabinozide cytarabine. Anticancer Drugs..

[219] Xu Q, Thompson JE, Carroll M (2005). mTOR regulates cell survival after etoposide treatment in primary AML cells. Blood..

[220] Perl AE, Kasner MT, Tsai DE, Vogl DT, Loren AW, Schuster SJ, Porter DL, Stadtmauer EA, Goldstein SC, Frey NV, Nasta SD, Hexner EO, Dierov JK, Swider CR, Bagg A, Gewirtz AM, Carroll M, Luger SM (2009). A phase I study of the mammalian target of rapamycin inhibitor sirolimus and MEC chemotherapy in relapsed and refractory acute myelogenous leukemia. Clin Cancer Res..

[221] Burnett AK, Das Gupta E, Knapper S, Khwaja A, Sweeney M, Kjeldsen L, Hawkins T, Betteridge SE, Cahalin P, Clark RE, Hills RK, Russell NH, UK NCRI AML Study Group (2018). Addition of the mammalian target of rapamycin inhibitor, everolimus, to consolidation therapy in acute myeloid leukemia: experience from the UK NCRI AML17 trial. Haematologica..

[222] Tiong IS, Tan P, McManus J, Cummings N, Sadawarte S, Catalano J, Hills R, Wei A (2018). Phase Ib study of the mTOR inhibitor everolimus with low dose cytarabine in elderly acute myeloid leukemia. Leuk Lymphoma..

[223] Rushworth SA, Murray MY, Zaitseva L, Bowles KM, MacEwan DJ (2014). Identification of Bruton's tyrosine kinase as a therapeutic target in acute myeloid leukemia. Blood..

[224] Cortes JE, Jonas BA, Graef T, Luan Y, Stein AS (2019). Clinical experience with ibrutinib alone or in combination with either cytarabine or azacitidine in patients with acute myeloid leukemia. Clin Lymphoma Myeloma Leuk.

[225] Nguyen LXT, Troadec E, Kalvala A, Kumar B, Hoang DH, Viola D, Zhang B, Nguyen DQ, Aldoss I, Ghoda L, Budde E, Pichiorri F, Rosen S, Forman SJ, Marcucci G, Pullarkat V (2019). The Bcl-2 inhibitor venetoclax inhibits Nrf2 antioxidant pathway activation induced by hypomethylating agents in AML. J Cell Physiol..

[226] Jones CL, Stevens BM, D'Alessandro A, Reisz JA, Culp-Hill R, Nemkov T (2018). Inhibition of amino acid metabolism selectively targets human leukemia stem cells. Cancer Cell.

[227] DiNardo CD, Pratz KW, Letai A, Jonas BA, Wei AH, Thirman M (2018). Safety and preliminary efficacy of venetoclax with decitabine or azacitidine in elderly patients with previously untreated acute myeloid leukaemia: a non-randomised, open-label, phase 1b study. Lancet Oncol..

[228] Niu X, Zhao J, Ma J, Xie C, Edwards H, Wang G, Caldwell JT, Xiang S, Zhang X, Chu R, Wang ZJ, Lin H, Taub JW, Ge Y (2016). Binding of released Bim to Mcl-1 is a mechanism of intrinsic resistance to ABT-199 which can be Overcome by combination with daunorubicin or cytarabine in AML cells. Clin Cancer Res..

[229] Wei AH, Strickland SA, Hou JZ, Fiedler W, Lin TL, Walter RB (2019). Venetoclax Combined With Low-Dose Cytarabine for Previously Untreated Patients With Acute Myeloid Leukemia: Results From a Phase Ib/II Study. J Clin Oncol..

[230] Karol SE, Alexander TB, Budhraja A, Pounds SB, Canavera K, Wang L, Wolf J, Klco JM, Mead PE, Das Gupta S, Kim SY, Salem AH, Palenski T, Lacayo NJ, Pui CH, Opferman JT, Rubnitz JE (2020). Venetoclax in combination with cytarabine with or without idarubicin in children with relapsed or refractory acute myeloid leukaemia: a phase 1, dose-escalation study. Lancet Oncol..

[231] Chyla B, Daver N, Doyle K, McKeegan E, Huang X, Ruvolo V, Wang Z, Chen K, Souers A, Leverson J, Potluri J, Boghaert E, Bhathena A, Konopleva M, Popovic R (2018). Genetic biomarkers of sensitivity and resistance to venetoclax monotherapy in patients with relapsed acute myeloid leukemia. Am J Hematol..

[232] Cathelin S, Sharon D, Subedi A, Cojocari D, Phillips DC, Leverson JD, MacBeth K, Nicolay B, Narayanaswamy R, Ronseaux S, Liu G, Chan SM (2018). Combination of Enasidenib and venetoclax shows superior anti-leukemic activity against IDH2 mutated AML in patient-derived xenograft models. Blood..

[233] Moujalled DM, Pomilio G, Ghiurau C, Ivey A, Salmon J, Rijal S, Macraild S, Zhang L, Teh TC, Tiong IS, Lan P, Chanrion M, Claperon A, Rocchetti F, Zichi A, Kraus-Berthier L, Wang Y, Halilovic E, Morris E, Colland F, Segal D, Huang D, Roberts AW, Maragno AL, Lessene G, Geneste O, Wei AH (2019). Combining BH3-mimetics to target both BCL-2 and MCL1 has potent activity in pre-clinical models of acute myeloid leukemia. Leukemia..

[234] Anstee NS, Bilardi RA, Ng AP, Xu Z, Robati M, Vandenberg CJ, Cory S (2019). Impact of elevated anti-apoptotic MCL-1 and BCL-2 on the development and treatment of MLL-AF9 AML in mice. Cell Death Differ..

[235] Cerella C, Gaigneaux A, Mazumder A, Lee JY, Saland E, Radogna F, Farge T, Vergez F, Récher C, Sarry JE, Kim KW, Shin HY, Dicato M, Diederich M (2017). Bcl-2 protein family expression pattern determines synergistic pro-apoptotic effects of BH3 mimetics with hemisynthetic cardiac glycoside UNBS1450 in acute myeloid leukemia. Leukemia..

[236] Wei Y, Kadia T, Tong W, Zhang M, Jia Y, Yang H, Hu Y, Tambaro FP, Viallet J, O'Brien S, Garcia-Manero G (2010). The combination of a histone deacetylase inhibitor with the Bcl-2 homology domain-3 mimetic GX15-070 has synergistic antileukemia activity by activating both apoptosis and autophagy. Clin Cancer Res..

[237] Cai T, Lorenzi PL, Rakheja D, Pontikos MA, Lodi A, Han L, Zhang Q, Ma H, Rahmani M, Bhagat TD, Horvath TD, DiNardo CD, Grant S, Tiziani S, Verma A, Konopleva M (2016). Gls Inhibitor CB-839 Modulates Cellular Metabolism in AML and Potently Suppresses AML Cell Growth When Combined with 5-Azacitidine. Blood..

[238] Gregory MA, Nemkov T, Reisz JA, Zaberezhnyy V, Hansen KC, DʼAlessandro A, et al. (2018). Glutaminase inhibition improves FLT3 inhibitor therapy for acute myeloid leukemia. Exp Hematol..

[239] Capizzi RL, Davis R, Powell B, Cuttner J, Ellison RR, Cooper MR, Dillman R, Major WB, Dupre E, McIntyre OR (1988). Synergy between high-dose cytarabine and asparaginase in the treatment of adults with refractory and relapsed acute myelogenous leukemia--a Cancer and Leukemia Group B Study. J Clin Oncol..

[240] Ahmed T, Holwerda S, Klepin HD, Isom S, Ellis LR, Lyerly S, Manuel M, Dralle S, Berenzon D, Powell BL, Pardee TS (2015). High dose cytarabine, mitoxantrone and l-asparaginase (HAMA) salvage for relapsed or refractory acute myeloid leukemia (AML) in the elderly. Leuk Res..

[241] Estañ MC, Calviño E, Calvo S, Guillén-Guío B, MeC B-A, de Blas E (2014). Apoptotic efficacy of etomoxir in human acute myeloid leukemia cells. Cooperation with arsenic trioxide and glycolytic inhibitors, and regulation by oxidative stress and protein kinase activities. Plos One.

[242] Tabe Y, Saitoh K, Yang H, Sekihara K, Yamatani K, Ruvolo V, Taka H, Kaga N, Kikkawa M, Arai H, Miida T, Andreeff M, Spagnuolo PA, Konopleva M (2018). Inhibition of FAO in AML co-cultured with BM adipocytes: mechanisms of survival and chemosensitization to cytarabine. Sci Rep..

[243] Wang F, Liu Z, Zeng J, Zhu H, Li J, Cheng X, Jiang T, Zhang L, Zhang C, Chen T, Liu T, Jia Y (2015). Metformin synergistically sensitizes FLT3-ITD-positive acute myeloid leukemia to sorafenib by promoting mTOR-mediated apoptosis and autophagy. Leuk Res..

[244] Sabnis HS, Bradley HL, Tripathi S, Yu WM, Tse W, Qu CK, Bunting KD (2016). Synergistic cell death in FLT3-ITD positive acute myeloid leukemia by combined treatment with metformin and 6-benzylthioinosine. Leuk Res..

[245] Yuan F, Cheng C, Xiao F, Liu H, Cao S, Zhou G (2020). Inhibition of mTORC1/P70S6K pathway by Metformin synergistically sensitizes Acute Myeloid Leukemia to Ara-C. Life Sci..

[246] Han L, Cavazos A, Baran N, Zhang Q, Kuruvilla VM, Gay JP, Feng N, Battula VL, Kantarjian HM, Daver NG, Marszalek JR, Andreeff M, Konopleva MY (2019). Mitochondrial OxPhos as survival mechanism of minimal residual AML cells after induction chemotherapy: survival benefit by complex I inhibition with IACS-010759. Blood..

[247] Pfefferle A, Mailloux RJ, Adjeitey CN, Harper ME (2013). Glutathionylation of UCP2 sensitizes drug resistant leukemia cells to chemotherapeutics. Biochim Biophys Acta..

[248] Douer D, Watkins K, Louie R, Weitz I, Mohrbacher A, Levine AM (2004). Treatment of acute myelogenous leukemia (non-APL) with intravenous Trisenox (arsenic trioxide) and ascorbic acid: preliminary results. Blood..

[249] Chau D, Ng K, Chan TS, Cheng YY, Fong B, Tam S (2015). Azacytidine sensitizes acute myeloid leukemia cells to arsenic trioxide by up-regulating the arsenic transporter aquaglyceroporin 9. J Hematol Oncol..

[250] Welch JS, Klco JM, Gao F, Procknow E, Uy GL, Stockerl-Goldstein KE, Abboud CN, Westervelt P, DiPersio JF, Hassan A, Cashen AF, Vij R (2011). Combination decitabine, arsenic trioxide, and ascorbic acid for the treatment of myelodysplastic syndrome and acute myeloid leukemia: a phase I study. Am J Hematol..

[251] Roboz GJ, Ritchie EK, Curcio T, Provenzano J, Carlin R, Samuel M, Wittenberg B, Mazumdar M, Christos PJ, Mathew S, Allen-Bard S, Feldman EJ (2008). Arsenic trioxide and low-dose cytarabine in older patients with untreated acute myeloid leukemia, excluding acute promyelocytic leukemia. Cancer..

[252] Burnett AK, Hills RK, Hunter A, Milligan D, Kell J, Wheatley K, Yin J, McMullin MF, Cahalin P, Craig J, Bowen D, Russell N (2011). The addition of arsenic trioxide to low-dose Ara-C in older patients with AML does not improve outcome. Leukemia..

[253] Wetzler M, Andrews C, Ford LA, Tighe S, Barcos M, Sait SN, Block AW, Nowak NJ, Baer MR, Wang ES, Baumann H (2011). Phase 1 study of arsenic trioxide, high-dose cytarabine, and idarubicin to down-regulate constitutive signal transducer and activator of transcription 3 activity in patients aged <60 years with acute myeloid leukemia. Cancer..

[254] Dembitz V, Lalic H, Ostojic A, Vrhovac R, Banfic H, Visnjic D (2015). The mechanism of synergistic effects of arsenic trioxide and rapamycin in acute myeloid leukemia cell lines lacking typical t(15;17) translocation. Int J Hematol..

[255] Liesveld JL, Rosell KE, Bechelli J, Lu C, Messina P, Mulford D, Ifthikharuddin JJ, Jordan CT, Phillips Ii GL (2011). Proteasome inhibition in myelodysplastic syndromes and acute myelogenous leukemia cell lines. Cancer Invest..

[256] Ganesan S, Alex AA, Chendamarai E, Balasundaram N, Palani HK, David S, Kulkarni U, Aiyaz M, Mugasimangalam R, Korula A, Abraham A, Srivastava A, Padua RA, Chomienne C, George B, Balasubramanian P, Mathews V (2016). Rationale and efficacy of proteasome inhibitor combined with arsenic trioxide in the treatment of acute promyelocytic leukemia. Leukemia..

[257] Kulkarni U, Ganesan S, Alex AA, Palani H, David S, Balasundaram N, Venkatraman A, Thenmozhi M, Jeyaseelan L, Korula A, Devasia A, Abraham A, Janet NB, Balasubramanian P, George B, Mathews V (2020). A phase II study evaluating the role of bortezomib in the management of relapsed acute promyelocytic leukemia treated upfront with arsenic trioxide. Cancer Med..

[258] Calviño E, Estañ MC, Simón GP, Sancho P, MeC B-A, de Blas E (2011). Increased apoptotic efficacy of lonidamine plus arsenic trioxide combination in human leukemia cells. Reactive oxygen species generation and defensive protein kinase (MEK/ERK, Akt/mTOR) modulation. Biochem Pharmacol..

[259] Emadi A, Sadowska M, Carter-Cooper B, Bhatnagar V, van der Merwe I, Levis MJ, Sausville EA, Lapidus RG (2015). Perturbation of cellular oxidative state induced by dichloroacetate and arsenic trioxide for treatment of acute myeloid leukemia. Leuk Res..

[260] Zheng PZ, Wang KK, Zhang QY, Huang QH, Du YZ, Zhang QH (2005). Systems analysis of transcriptome and proteome in retinoic acid/arsenic trioxide-induced cell differentiation/apoptosis of promyelocytic leukemia. Proc Natl Acad Sci U S A..

[261] Lo-Coco F, Avvisati G, Vignetti M, Thiede C, Orlando SM, Iacobelli S, Ferrara F, Fazi P, Cicconi L, di Bona E, Specchia G, Sica S, Divona M, Levis A, Fiedler W, Cerqui E, Breccia M, Fioritoni G, Salih HR, Cazzola M, Melillo L, Carella AM, Brandts CH, Morra E, von Lilienfeld-Toal M, Hertenstein B, Wattad M, Lübbert M, Hänel M, Schmitz N, Link H, Kropp MG, Rambaldi A, la Nasa G, Luppi M, Ciceri F, Finizio O, Venditti A, Fabbiano F, Döhner K, Sauer M, Ganser A, Amadori S, Mandelli F, Döhner H, Ehninger G, Schlenk RF, Platzbecker U (2013). Retinoic acid and arsenic trioxide for acute promyelocytic leukemia. N Engl J Med..

[262] Rotin LE, Gronda M, Maclean N, Lin F-H, Wrana J, Datti A, Barber DL, Moran MF, Minden MD, Slassi M, Schimmer AD (2014). Ibrutinib sensitizes AML cells to ROS inducers via a BTK-independent mechanism. Blood..

[263] Liu Y, Chen F, Wang S, Guo X, Shi P, Wang W, Xu B (2013). Low-dose triptolide in combination with idarubicin induces apoptosis in AML leukemic stem-like KG1a cell line by modulation of the intrinsic and extrinsic factors. Cell Death Dis..

[264] Yaseen A, Chen S, Hock S, Rosato R, Dent P, Dai Y, Grant S (2012). Resveratrol sensitizes acute myelogenous leukemia cells to histone deacetylase inhibitors through reactive oxygen species-mediated activation of the extrinsic apoptotic pathway. Mol Pharmacol..

[265] Walter RB, Pirga JL, Cronk MR, Mayer S, Appelbaum FR, Banker DE (2005). PK11195, a peripheral benzodiazepine receptor (pBR) ligand, broadly blocks drug efflux to chemosensitize leukemia and myeloma cells by a pBR-independent, direct transporter-modulating mechanism. Blood..

[266] Bosnjak M, Ristic B, Arsikin K, Mircic A, Suzin-Zivkovic V, Perovic V, Bogdanovic A, Paunovic V, Markovic I, Bumbasirevic V, Trajkovic V, Harhaji-Trajkovic L (2014). Inhibition of mTOR-dependent autophagy sensitizes leukemic cells to cytarabine-induced apoptotic death. PLoS One..

[267] Kim Y, Eom JI, Jeung HK, Jang JE, Kim JS, Cheong JW, Kim YS, Min YH (2015). Induction of cytosine arabinoside-resistant human myeloid leukemia cell death through autophagy regulation by hydroxychloroquine. Biomed Pharmacother..

[268] Cheong JW, Kim Y, Eom JI, Jeung HK, Min YH (2016). Enhanced autophagy in cytarabine arabinoside-resistant U937 leukemia cells and its potential as a target for overcoming resistance. Mol Med Rep..

[269] Tanios R, Bekdash A, Kassab E, Stone E, Georgiou G, Frankel AE, Abi-Habib RJ (2013). Human recombinant arginase I(Co)-PEG5000 [HuArgI(Co)-PEG5000]-induced arginine depletion is selectively cytotoxic to human acute myeloid leukemia cells. Leuk Res..

[270] Torgersen ML, Engedal N, Bøe SO, Hokland P, Simonsen A (2013). Targeting autophagy potentiates the apoptotic effect of histone deacetylase inhibitors in t(8;21) AML cells. Blood..

[271] Nawrocki ST, Han Y, Visconte V, Przychodzen B, Espitia CM, Phillips J, Anwer F, Advani A, Carraway HE, Kelly KR, Sekeres MA, Maciejewski JP, Carew JS (2019). The novel autophagy inhibitor ROC-325 augments the antileukemic activity of azacitidine. Leukemia..

[272] Bhattacharya S, Piya S, McQueen T, Konopleva M, Andreeff M, Borthakur G (2017). Inhibition of Unc-1 like autophagy activating kinase 1 (ULK1) is highly synergistic with chemotherapy and Bcl2 inhibition in acute myeloid leukemia (AML). Blood..

[273] Bhattacharya S, Piya S, Zhang Q, Baran N, McQueen T, Davis RE, Cosford N, Konopleva MY, Andreeff M, Borthakur G (2018). Targeting autophagy kinase ULK1 can reverse Bcl2 inhibitor (ABT-199) induced autophagy to overcome acquired resistance in acute myeloid leukemia. Blood..

[274] Qiu L, Zhou G, Cao S (2020). Targeted inhibition of ULK1 enhances daunorubicin sensitivity in acute myeloid leukemia. Life Sci..

[275] Stewart HJS, Chaudry S, Crichlow A, Luiling Feilding F, Chevassut TJT (2018). BET Inhibition suppresses S100A8 and S100A9 expression in acute myeloid leukemia cells and synergises with daunorubicin in causing cell death. Bone Marrow Res..

[276] Herrmann H, Blatt K, Shi J, Gleixner KV, Cerny-Reiterer S, Müllauer L, Vakoc CR, Sperr WR, Horny HP, Bradner JE, Zuber J, Valent P (2012). Small-molecule inhibition of BRD4 as a new potent approach to eliminate leukemic stem- and progenitor cells in acute myeloid leukemia AML. Oncotarget..

[277] Karjalainen R, Liu M, Kumar A, Parsons A, He L, Malani DR, Kontro M, Porkka K, Heckman CA (2018). Combined targeting of BET family proteins and BCL2 Is synergistic in acute myeloid leukemia cells overexpressing *S100A8* and *S100A9*. Blood..

[278] Ouchida AT, Li Y, Geng J, Najafov A, Ofengeim D, Sun X, Yu Q, Yuan J (2018). Synergistic effect of a novel autophagy inhibitor and quizartinib enhances cancer cell death. Cell Death Dis..

[279] Ha YN, Song S, Orlikova-Boyer B, Cerella C, Christov C, Kijjoa A, et al. Petromurin C induces protective autophagy and apoptosis in FLT3-ITD-positive AML: synergy with gilteritinib. Mar Drugs. 2020;18(1):57. 10.3390/md18010057 PMC7024157 31963113

[280] Bai H, Cao Z, Deng C, Zhou L, Wang C (2012). miR-181a sensitizes resistant leukaemia HL-60/Ara-C cells to Ara-C by inducing apoptosis. J Cancer Res Clin Oncol..

[281] Huang X, Schwind S, Santhanam R, Eisfeld AK, Chiang CL, Lankenau M, Yu B, Hoellerbauer P, Jin Y, Tarighat SS, Khalife J, Walker A, Perrotti D, Bloomfield CD, Wang H, Lee RJ, Lee LJ, Marcucci G (2016). Targeting the RAS/MAPK pathway with miR-181a in acute myeloid leukemia. Oncotarget..

[282] Lu F, Zhang J, Ji M, Li P, Du Y, Wang H (2014). miR-181b increases drug sensitivity in acute myeloid leukemia via targeting HMGB1 and Mcl-1. Int J Oncol..

[283] Gao SM, Chen C, Wu J, Tan Y, Yu K, Xing CY, Ye A, Yin L, Jiang L (2010). Synergistic apoptosis induction in leukemic cells by miR-15a/16-1 and arsenic trioxide. Biochem Biophys Res Commun..

[284] Liu Y, Lei P, Qiao H, Sun K, Lu X, Bao F, Yu R, Lian C, Li Y, Chen W, Xue F (2019). miR-9 Enhances the chemosensitivity of AML cells to daunorubicin by targeting the EIF5A2/MCL-1 axis. Int J Biol Sci..

[285] Garzon R, Pichiorri F, Marcucci G, Kornblau S, Andreeff M, Croce C (2007). MiRNA-29b targets MCL-1 and is down-regulated in chemotherapy-resistant acute myeloid leukemia (AML). Blood..

[286] Huang X, Schwind S, Yu B, Santhanam R, Wang H, Hoellerbauer P, Mims A, Klisovic R, Walker AR, Chan KK, Blum W, Perrotti D, Byrd JC, Bloomfield CD, Caligiuri MA, Lee RJ, Garzon R, Muthusamy N, Lee LJ, Marcucci G (2013). Targeted delivery of microRNA-29b by transferrin-conjugated anionic lipopolyplex nanoparticles: a novel therapeutic strategy in acute myeloid leukemia. Clin Cancer Res..

[287] Gocek E, Wang X, Liu X, Liu CG, Studzinski GP (2011). MicroRNA-32 upregulation by 1,25-dihydroxyvitamin D3 in human myeloid leukemia cells leads to Bim targeting and inhibition of AraC-induced apoptosis. Cancer Res..

[288] Kurtz SE, Eide CA, Kaempf A, Khanna V, Savage SL, Rofelty A, English I, Ho H, Pandya R, Bolosky WJ, Poon H, Deininger MW, Collins R, Swords RT, Watts J, Pollyea DA, Medeiros BC, Traer E, Tognon CE, Mori M, Druker BJ, Tyner JW (2017). Molecularly targeted drug combinations demonstrate selective effectiveness for myeloid- and lymphoid-derived hematologic malignancies. Proc Natl Acad Sci U S A..

[289] Chang E, Ganguly S, Rajkhowa T, Gocke CD, Levis M, Konig H (2016). The combination of FLT3 and DNA methyltransferase inhibition is synergistically cytotoxic to FLT3/ITD acute myeloid leukemia cells. Leukemia..

[290] Ramaswamy S (2007). Rational design of cancer-drug combinations. N Engl J Med..

[291] Si W, Shen J, Zheng H, Fan W (2019). The role and mechanisms of action of microRNAs in cancer drug resistance. Clin Epigenetics..

[292] Auberger P, Puissant A (2017). Autophagy, a key mechanism of oncogenesis and resistance in leukemia. Blood..

